# Zinc Single Atom Confinement Effects on Catalysis in 1T-Phase Molybdenum Disulfide

**DOI:** 10.1021/acsnano.2c09918

**Published:** 2023-01-11

**Authors:** Sabrina M. Younan, Zhida Li, XingXu Yan, Dong He, Wenhui Hu, Nino Demetrashvili, Gabriella Trulson, Audrey Washington, Xiangheng Xiao, Xiaoqing Pan, Jier Huang, Jing Gu

**Affiliations:** † Department of Chemistry and Biochemistry, San Diego State University, 5500 Campanile DriveSan Diego, California92182, United States; % State Key Laboratory of Urban Water Resource and Environment, School of Civil and Environmental Engineering, Harbin Institute of Technology, Shenzhen518055, China; § Department of Materials Science and Engineering, University of California, Irvine, California92697, United States; ∥ Department of Physics and Astronomy, University of California, Irvine, California92697, United States; ⊥ Department of Physics, Wuhan University, Wuhan430072, China; # Department of Chemistry, Marquette University, Milwaukee, Wisconsin53201, United States

**Keywords:** confinement effects, heterogeneous catalysis, intercalations, layered compounds, single atom catalysis, molybdenum disulfide, two-dimensional materials

## Abstract

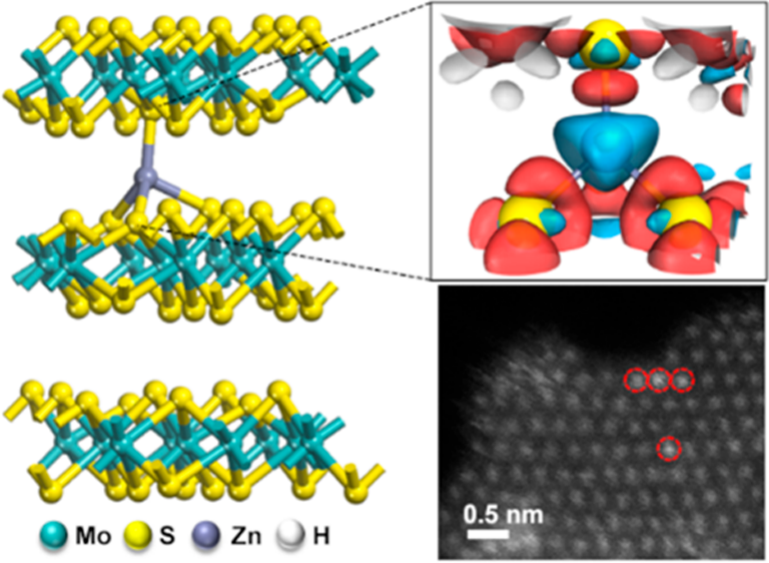

Active sites are atomic sites within catalysts that drive reactions and are essential for catalysis. Spatially confining guest metals within active site microenvironments has been predicted to improve catalytic activity by altering the electronic states of active sites. Using the hydrogen evolution reaction (HER) as a model reaction, we show that intercalating zinc single atoms between layers of 1T-MoS_2_ (Zn SAs/1T-MoS_2_) enhances HER performance by decreasing the overpotential, charge transfer resistance, and kinetic barrier. The confined Zn atoms tetrahedrally coordinate to basal sulfur (S) atoms and expand the interlayer spacing of 1T-MoS_2_ by ∼3.4%. Under confinement, the Zn SAs donate electrons to coordinated S atoms, which lowers the free energy barrier of H* adsorption–desorption and enhances HER kinetics. In this work, which is applicable to all types of catalytic reactions and layered materials, HER performance is enhanced by controlling the coordination geometry and electronic states of transition metals confined within active-site microenvironments.

## Introduction

Hydrogen (H_2_) is recognized as an essential green energy carrier that is widely used as a chemical feedstock in petroleum refinement, fertilizer production, and as a fuel source for electricity/heat generation.^1^ As of 2021, 95% of H_2_ produced in the United States is generated by steam–methane reformation.^2^ In addition to being energy-intensive, this centralized process increases CO_2_ emissions and requires large power plants that are expensive to build. Consequently, this method of H_2_ generation is not accessible to countries that lack these resources.^3^

Luckily, alternative pathways to H_2_ generation exist.^4^ Using electrochemical methods to drive the H_2_ evolution reaction (HER) provides a sustainable, decentralized alternative by requiring only water to yield H_2_ with high purity.^5,6^ As such, it circumvents the challenges of steam–methane reformation and enables countries with limited resources to become more self-reliant. Before electrochemical H_2_ generation can be implemented in society, critical obstacles still need to be overcome. Primarily, electrocatalysts that are cost-effective, highly efficient, and durable must be developed to replace the precious metal electrocatalysts currently employed in commercialized electrolyzers.^7−9^ Since the performance of electrocatalysts depends on the nature of their active sites, methods that maximize active site performance in the next generation of electrocatalysts need to be established.

Theoretical studies have revealed that spatially confining microenvironments (the local coordination environment and electronic states of active sites) within catalysts can enhance the catalyst’s activity by modulating the frontier orbital energies and adsorption–desorption energies of active sites.^10,11^ Electronic properties of confined active sites are directly influenced by their coordination environment, which in turn alters the adsorption energetics of reaction intermediates and catalytic activity/selectivity.^12^ Therefore, the relationship among active sites, local microenvironments, and confined species dictates catalytic performance. However, designing a prototype system that enables understanding of catalytic confinement effects at the atomic level remains challenging.

Single-atom catalysts (SACs), in which single atoms (SAs) are stabilized within supporting substrates by either adsorbing to the substrate’s basal plane or substituting atoms within the substrate’s lattice, offer an ideal prototype for the investigation of confinement catalysis. Along with demonstrating a superior catalytic performance compared to nanoparticles and nanoclusters in traditional metal catalysts, SACs exhibit flexibility with respect to crystallinity, coordination number, and electronic structures.^13^ Likewise, when employing noble metal SAs, SACs require significantly smaller quantities of noble metals to achieve competitive catalytic performance to produce solar fuels and industrial chemicals.

To understand the confinement effects between catalysts and their microenvironments, identifying a paradigmatic support material is crucial. Commonly employed scaffolds that provide spatial confinement include channels in carbon nanotubes (CNTs) and porous sites in zeolites and metal organic frameworks (MOFs).^12,14^ In these cases, methods such as doping the catalyst with nonmetals, forming bimetallic active sites, and synthetically inducing an anisotropic catalyst surface have all been shown to enhance the catalytic activity.^15−17^ However, both zero-dimensional (0D) nanocavities in zeolites/MOFs and one-dimensional (1D) nanocavities in CNTs suffer from major disadvantages such as complex structural and chemical composition.^12,14^ These complexities create an uneven environment surrounding the active sites and make understanding confinement effects very difficult at the microscopic level.^12,14^

Compared to their three-dimensional (3D) counterparts, two-dimensional (2D) materials exhibit well-defined layered structures, a variety of polymorphs, and tunable geometric and electronic properties. Computational studies have predicted the interactions between active sites and guest species confined within their local microenvironments to heavily influence catalytic activity.^18−20^ Yet, experimental evidence of 2D materials other than carbon-based 2D materials is limited.^21^ For these reasons, exploring other 2D materials for confinement studies would provide an ideal platform to understand how confining guest species near active sites located within the substrate’s interlayer spacing influences catalytic performance.

HER is an ideal model reaction for confinement studies due to the fast kinetics of proton (H^+^) diffusion that occurs between layers of 2D materials.^14^ Customarily, the HER activity of HER catalysts is evaluated using hydrogen adsorption free energies (Δ*G*_H*_). Both nonmetal and transition metal HER catalysts follow the same trend, where maximum HER activity is achieved at around Δ*G*_H*_ = 0 eV.^7,22^ In the continuous search for Earth-abundant catalysts, MoS_2_ serves as a role model in HER catalysis.^7−9^ For decades, its activity has been considered limited due to the extremely high energy of proton adsorption on the basal plane of semiconducting 2H-MoS_2_ (Δ*G*_H*_ = 1.92 eV). This changed when theoretical calculations revealed the extremely thermoneutral nature of the edge sites in 2H-MoS_2_ (Δ*G*_H*_ = 0.08 eV).^23^ Volcano plots published in the literature that are used to access HER activities of metal nanoparticles and other HER catalysts have demonstrated that, compared to other commonly employed nonprecious HER catalysts, molybdenum dichalcogenides maintain a Δ*G*_H*_ nearest to zero.^7,9,24,25^ Therefore, MoS_2_ is considered to be an ideal candidate because of the fast HER kinetics that it sustains. The best performing HER catalysts are precious metals which are rare and expensive, such as platinum (Pt). In contrast, MoS_2_ is composed of Earth-abundant elements and thus is much less expensive and more feasible at scale. For these reasons, MoS_2_ is considered to be more advantageous than other high-performance HER catalysts.

Various strategies have been employed to expose more active edge sites in 2H-MoS_2_.^26,27^ For instance, Wang et al. employed a mild H_2_O_2_ chemical etching strategy to investigate the impact of both the concentration and distribution of S vacancies in MoS_2_ on HER activity.^28^ The results suggest that the homogeneous distribution of single S-vacancies throughout the MoS_2_ nanosheet surface achieves optimal HER performance, as demonstrated by the 48 mV/dec Tafel slope and 131 mV overpotential reported. Subsequently, the direction of MoS_2_-based HER research shifted with the discovery of the metallic 1T-phase of MoS_2_, due to the higher density of active sites available along the basal plane of 1T-MoS_2_.^29,30^

Compared to the trigonal prismatic 2H-phase of MoS_2_, 1T-MoS_2_ layers feature well-defined octahedral symmetry, which increases the exposure of surface active sites for enhanced catalytic performance.^31^ Furthermore, metallic 1T-MoS_2_ exhibits exceptional charge transport properties compared to its 2H-phase semiconducting analogue, thus enabling further exposure of active sites, at which surface reactions take place.^32,33^ While 2H-MoS_2_ is known to be the more thermodynamically stable phase in nature, intercalation of SAs between layers of MoS_2_ enables MoS_2_ to remain stable in the 1T-phase.^34^ In addition, the distinct, local atomic environment and uniform chemical nature of SAs offer advantages of distinguished activity, selectivity, and stability studies for the HER.

Thus far, only a few catalytic confinement studies have utilized MoS_2_ as the host material. For example, Chen et al. intercalated Pt nanoparticles within the van der Waals gaps of bulk MoS_2_ and discovered that confinement not only suppressed the aggregation of Pt nanoparticles but also facilitated the transfer of H_3_O^+^ during HER.^35^ Likewise, Luo et al. inserted Co(OH)_2_ nanoparticles between layers of bulk MoS_2_ to improve the HER performance of MoS_2_ under alkaline media.^36^ Unfortunately, previous confinement studies suffer from either utilization of precious metals or difficulty with controlling the size and structure of intercalated species, resulting in an unsystematic study of confinement effects.

Other works have investigated the catalytic effects of modifying 1T-MoS_2_ with first-row transition metals. Huang et al. discovered that hydrothermally synthesizing 1T-MoS_2_ with Fe, Co, and Ni enhances HER activity in alkaline media by doping the guest metals into the 1T-MoS_2_ lattice in a 1:6 X:Mo ratio (X = Fe, Co, or Ni).^37^ Li et al. reported the ability to enhance the HER performance of 1T-MoS_2_ by either substituting lattice sites with copper (Cu) SAs or adsorbing Cu SAs along the 1T-MoS_2_ basal plane.^38^ Each of these methods of stabilizing Cu SAs was achieved by employing syringe injection and hydrothermal synthetic methods, respectively. Meanwhile, in a volcano plot reported by Deng et al., Zn demonstrated a Δ*G*_H*_ value near 0 eV, indicating that it is one of the few nonprecious metals that may be able to modify the HER activity of MoS_2_.^39^ Therefore, Zn SAs were selected as the guest intercalants in this study to investigate the effects of nonprecious metal confinement effects on HER catalysis.

In this work, Zn SAs were intercalated within the interlayer spacing of 1T-MoS_2_ (Zn SAs/1T-MoS_2_) via syringe injection into hydrothermally synthesized 1T-MoS_2_ (see the Methods). The results reported herein show that the confined SAs maintain a Zn^2+^ oxidation state and expand the interlayer spacing of 1T-MoS_2_ by ∼3.4% (0.022 nm). Changes in catalytic performance during HER were monitored electrochemically under acidic conditions, where a decrease in overpotential (1T-MoS_2_ = 265 mV; Zn SAs/1T-MoS_2_ = 177 mV) and charge transport limitations (1T-MoS_2_ = 106.4 mV/dec; Zn SAs/1T-MoS_2_ = 84.9 mV/dec) were observed when Zn SAs were intercalated between 1T-MoS_2_ layers. The experimental findings were further confirmed computationally using proton adsorption energies predicted from first-principles density functional theory (DFT) and partial density of states (PDOS) plots. In addition to facilitating HER kinetics, the spatial confinement of Zn SAs was predicted to enhance interactions between protons and the microenvironments of nearby active sites within which they are confined within.

## Results and Discussion

### Adsorption of Zinc Single Atoms along the Basal Plane of 1T-MoS_2_

**Figure 1:**
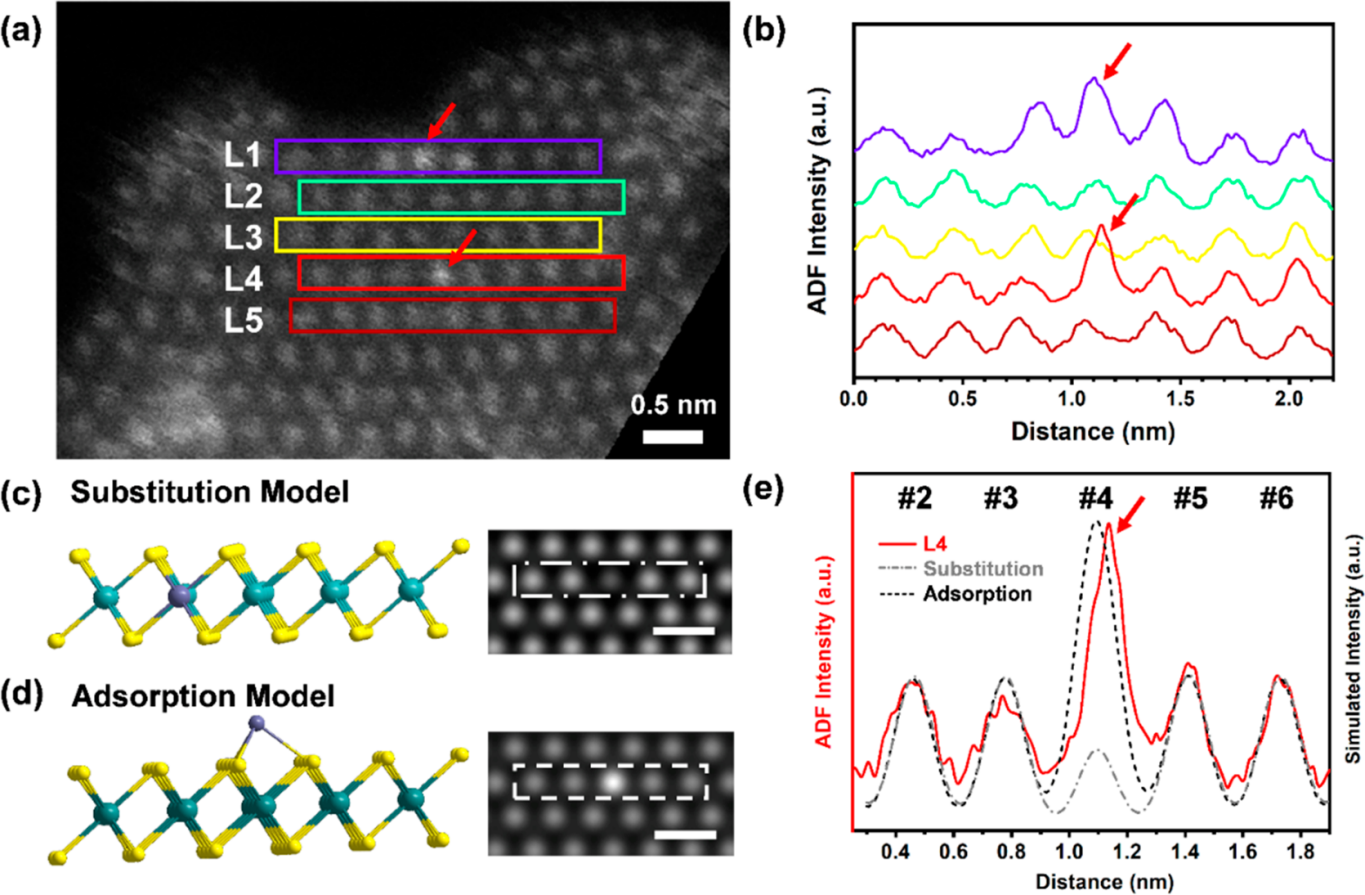
HAADF-STEM characterization and quantitative identification of Zn SAs adsorption to the basal plane of 1T-MoS_2_. (a) Atomically resolved HAADF-STEM image of the monolayer sample at 60 keV. (b) Intensity profiles taken along five adjacent lines (L1–L5) indicated by the different colored rectangles in (a). Red arrows point to the locations of the Zn SAs. (c) Side view of the atomic structure of the Zn substitution model and the simulated HAADF-STEM image. The yellow, green, and purple atoms represent S, Mo, and Zn, respectively. (d) Side view of the atomic structure of the Zn adsorption model and the simulated HAADF-STEM image. (e) Comparison of intensity profiles from the experimental image and two simulated images of the replaced and adsorbed models, respectively. All scale bars in (a, c, d) are 0.5 nm.

SAs may be stabilized on appropriate substrates by either substituting lattice vacancies or adsorbing to the substrate’s basal plane. In this work, high-angle annular dark-field scanning transmission electron microscopy (HAADF-STEM) was used to identify the positions occupied by Zn SAs in the 1T-MoS_2_ lattice (Figures 1, S1). In the HAADF-STEM image shown in Figure 1a, the intensity of each lattice site is correlated to the atomic number of elements occupying the lattice site.^40^ If the Zn SAs substitute Mo lattice sites, the lattice vacancies occupied by Zn SAs will appear darker since Zn (Z_Zn_ = 30) has a lower atomic number than Mo (Z_Mo_ = 42). Instead, brighter spots are observed, indicating that the Zn SAs adsorb to the basal plane and occupy sites above the Mo atoms. Additional evidence that the Zn SAs adsorb to the basal plane of 1T-MoS_2_ is shown by the HAADF-STEM images provided in Figure S1 (designated with red circles).

Line profiles taken from Figure 1a (labeled L1–L5) show an increase in annular dark field (ADF) intensity for lattice site positions with observably brighter intensities in L1 and L4 (Figure 1b). For comparison, line profiles extrapolated from the simulated models of substitution (Figure 1c) and adsorption (Figure 1d) were plotted against the experimental ADF intensity of L4 (Figure 1e). The results show that while the ADF intensity of L4 (1.95 au) does not match that of the simulated substitution model (0.52 au), it corresponds well with the ADF intensity of the simulated adsorption model (1.95 au). These results further confirm that the Zn SAs adsorb to the basal plane of 1T-MoS_2_.

HAADF-STEM images collected under continuous electron beam irradiation revealed the ability to trigger the migration of Zn SAs across the 1T-MoS_2_ basal plane (Figure S2). When the electron beam was focused on the same region for 109 s, the Zn atom located in L4 (*t* = 0 s, designated with a red arrow in Figure S2a) jumps to L5 (*t* = 109 s, Figure S2b). After 203 s, only one of the four Zn atoms (designated by the yellow arrow) present remains in the same position, while the other three Zn atoms migrate out of the scope area (Figure S2c). These observations further confirm that rather than substituting into the lattice, the Zn SAs adsorb to the basal plane of 1T-MoS_2_. It is worth mentioning that the dynamic movement of SAs under electron beam conditions is well-known.^41−43^ It would be intriguing to understand if the dynamics of SAs persist under catalytic conditions. However, a spectroscopic or microscopic technique needs to be developed that can confirm the dynamics of SAs while supplying the minimum amount of external energy required to trigger their movements.

### Expansion of 1T-MoS_2_ Lattice Fringe Spacing by Intercalated Zinc Single Atoms

**Figure 2:**
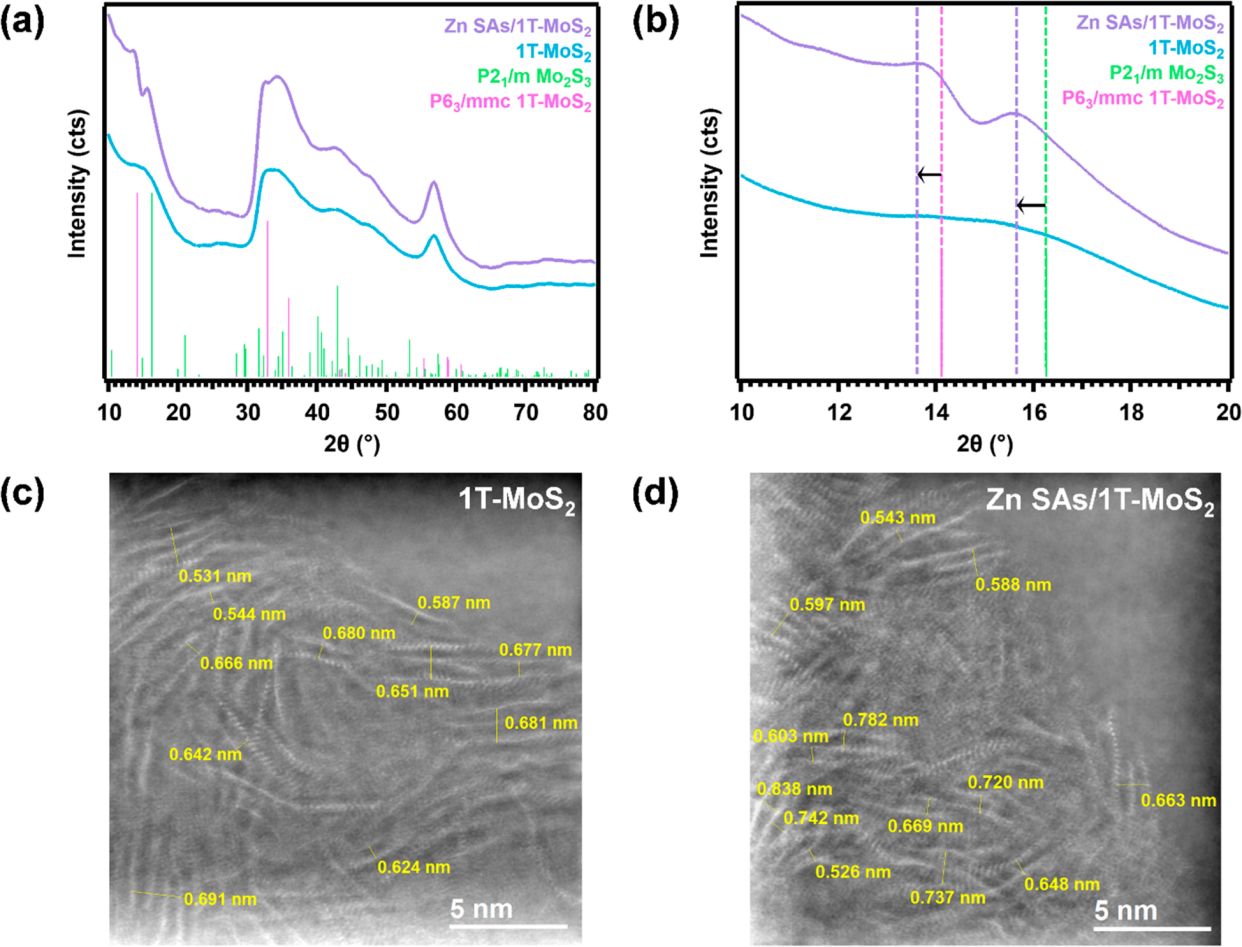
XRD and HRTEM analysis of 1T-MoS_2_ lattice expansion induced by intercalated Zn SAs. (a) Zn SAs/1T-MoS_2_ (purple) and 1T-MoS_2_ (blue) XRD patterns. (b) Zoomed in the region of XRD patterns to highlight shifts observed for the (001) and (101̅) planes in 1T-MoS_2_ and Mo_2_S_3_, respectively. (c) 1T-MoS_2_ HRTEM image. (d) Zn SAs/1T-MoS_2_ HRTEM image. Experimental fringe spacings are colored yellow.

By convention, intercalation reactions are characterized by the expansion of a layered substrate’s crystal lattice along the *c* axis.^44^ Expansion of 1T-MoS_2_’s interlayer spacing was evidenced by visible peak shifts in the X-ray diffraction (XRD) patterns of Zn SAs/1T-MoS_2_ compared to that of 1T-MoS_2_ (Figures 2a, 2b). Peak broadening observed in the diffraction pattern of bulk Zn SAs/1T-MoS_2_ compared to that of 1T-MoS_2_ arises from the overlap of hexagonal 1T-MoS_2_ (*P*6_3_/*mmc*; PDF no. 75-1539) and monoclinic Mo_2_S_3_ (*P*2_1_/*m*; PDF no. 72-0821). Mo_2_S_3_ consists of molecular Mo–S chains intercalated between 1T′-MoS_2_ layers (Figure S3).^45^ Interestingly, the peak overlap observed in 1T-MoS_2_ splits into two separate peaks in Zn SAs/1T-MoS_2_: the (001) plane bisecting the interlayer spacing of 1T-MoS_2_ and the (101̅) plane bisecting the Mo–S chains that intercalate the 1T’-MoS_2_ layers in Mo_2_S_3_. The diffraction peak indexed to the (001) plane of 1T-MoS_2_ shifts from 14.07° (0.629 nm) to 13.59° (0.651 nm) when Zn SAs are present, which corresponds to an ∼3.4% (0.022 nm) increase in 1T-MoS_2_’s interlayer spacing. Likewise, the peak indexed to the (101̅) plane of Mo_2_S_3_ shifts from 16.28° (0.544 nm) to 15.72° (0.563 nm) in Zn SAs/1T-MoS_2_ and also corresponds to an ∼3.4% (0.019 nm) expansion. DFT calculations reported in the literature have predicted a decreased diffusion energy barrier for SA migration between 2D layers as the interlayer spacing increases.^46,47^ Here, the interlayer spacing of 1T-MoS_2_ expands to accommodate the occupation of the interlayer lattice sites by Zn SAs.

Comparing the distance between lattice fringes in 1T-MoS_2_ to those found in Zn SAs/1T-MoS_2_ (Figure 2d) generates similar results. Images produced by high resolution TEM (HRTEM) with the electron beam aligned parallel to the 1T-MoS_2_ basal plane show an average distance of 0.632 ± 0.06 nm for lattice fringes found in 1T-MoS_2_ (Figure 2c). Lattice fringes present in Zn SAs/1T-MoS_2_ yield an average distance of 0.661 ± 0.09 nm (Figure 2d), which is roughly 0.029 nm larger than the 1T-MoS_2_ lattice fringes observed when Zn SAs are absent. This result corresponds to a 4.6% average increase in 1T-MoS_2_’s interlayer spacing. Altogether, the XRD and HRTEM results both suggest that intercalating Zn SAs expands 1T-MoS_2_’s interlayer spacing.

### Structural Characterization of Zn SAs/1T-MoS_2_

The Zn SAs/1T-MoS_2_ was analyzed by STEM and energy-dispersive X-ray (EDX) spectroscopy to atomically visualize the layer geometry and confirm the elemental components present (Figure S4). The resulting elemental maps of Zn, Mo, and S confirm the presence of these atoms in Zn SAs/1T-MoS_2_ and demonstrate the uniform distribution of Zn SAs throughout the substrate (Figures S4c, S4e, and S 4f). Correspondingly, the octahedral coordination of Mo and S atoms displayed throughout the Moiré patterns of Zn SAs/1T-MoS_2_ confirms the presence of the 1T phase of MoS_2_ (Figures S4a and S 4d).

To understand how the confinement of Zn SAs impacts the electronic structure of 1T-MoS_2_, X-ray photoelectron spectroscopy (XPS) was employed to compare the elemental and chemical compositions of Zn SAs/1T-MoS_2_ and 1T-MoS_2_ (Figure S5, Table S1). Peaks identified in the Zn 2p spectrum of Zn SAs/1T-MoS_2_ correspond to the presence of Zn^2+^ and confirm that the SAs retain their Zn^2+^ oxidation state after intercalation (Figure S5g).^48^ Deconvolution of peaks in the Mo 3d, S 2s (Figures S5a,b), and S 2p (Figures S5c,d) spectra produces two sets of doublet peaks, in which the set of peaks at lower binding energies (shown in purple) are assigned to Mo^4+^ and S^2–^ oxidation states in 1T-MoS_2_.^48^ The sets of doublet peaks at higher binding energies (shown in green) correspond to unsaturated Mo^5/6+^ and S^2–^ oxidation states and indicate the presence of a nonstoichiometric MoS_*x*_ species along the surface of each sample.^48^ Consequently, while the majority of both samples consists of stoichiometric 1T-MoS_2_, a portion of MoS_*x*_ exists near the catalyst surface.^49^ Compared to 1T-MoS_2_, Zn SAs/1T-MoS_2_ displays downshifts in binding energies of up to 0.19 eV. Downshifts of up to 0.10 eV fall within the step size (0.10 eV) employed during XPS analysis and as such are considered negligible. However, peak shifts exceeding the step size employed are observed for the S 2p doublet assigned to MoS_*x*_ (Figures S5c,d). This shift to lower binding energies indicates that the Zn SAs donate electrons to the S atoms of MoS_*x*_.^50^ This electron donation likely occurs to facilitate the stabilization of Zn SAs within the substrate’s interlayer spacing. Further evidence of the lack of structural changes to 1T-MoS_2_ upon intercalation of Zn SAs is provided by the FTIR spectra, which also demonstrates negligible chemical and electronic structure changes (Figure S6).

Next, Raman spectroscopy was employed to explore key structural details, such as lattice strain and vacancy defects. The Raman spectra for 1T-MoS_2_ and Zn SAs/1T-MoS_2_ are displayed in Figure S7 with experimental peak positions listed in Table S2. The existence of the 1T-phase in both samples is confirmed by the presence of transverse acoustic (TA) and longitudinal acoustic (LA) phonon modes at the M point of the first Brillouin zone and J_1_, J_2_, E_1g_, and J_3_ phonon modes. The negligible difference in the 1T-MoS_2_ and Zn SAs/1T-MoS_2_ peak positions provides further evidence that the lattice structure of 1T-MoS_2_ remains intact in the presence of Zn SAs.^51^

To compare differences in the magnetic properties and the amount of S vacancies, Zn SAs/1T-MoS_2_ and 1T-MoS_2_ were both evaluated by electron paramagnetic resonance (EPR) spectroscopy. Additionally, 2H-MoS_2_ was evaluated and treated as a control during EPR analysis. The EPR spectrum of 2H-MoS_2_ displays a narrow line shape and isotropic *g*-value (*g* = 2.005) that are attributed to dangling Mo–S bonds generated by S vacancies (Figure S8a).^25,52^ In contrast, 1T-MoS_2_ (Figure S8b) and Zn SAs/1T-MoS_2_ (Figure S8c) both produce complex EPR spectra in which more than one paramagnetic center is present. Since 1T-MoS_2_ and Zn SAs/1T-MoS_2_ yield similar *g*-values, the following assignments of paramagnetic centers apply to both samples. Direct correlation of the specific *g*-values each paramagnetic center is assigned to is provided in Table S3. The first paramagnetic center identified corresponds to paramagnetic S atoms in short chains (*g* = 2.042) and electron hole centers localized on S atoms (*g* ≈ 2.026).^53^ The second paramagnetic center, which generates *g*-values between 1.932 to 1.959 and 2.017 and 2.019, is assigned to Mo^5+^ species coordinated to S atoms.^54−56^ The existence of Mo^5+^ species corresponds to the presence of Mo species at higher oxidation states (Mo^5/6+^) in the XPS spectra (Figure S5) and is a result of local structural defects in 1T-MoS_2_ that give rise to under-coordinated Mo atoms within the substrate.^57^ The signals observed for *g*-values between 1.993 to 2.005 are ascribed to S–Mo^5+^ defects and dangling Mo–S bonds generated by S vacancies, respectively.^52,57^ The negligible difference in signal intensity of 1T-MoS_2_ from that of Zn SAs/1T-MoS_2_ indicates that the concentration of S vacancies is similar in both samples.^25,52,58^ Therefore, the influence of S vacancies on the catalytic performance of Zn SAs/1T-MoS_2_ is excluded.

### Coordination Environment and Valence States of Zn SAs/1T-MoS_2_

**Figure 3:**
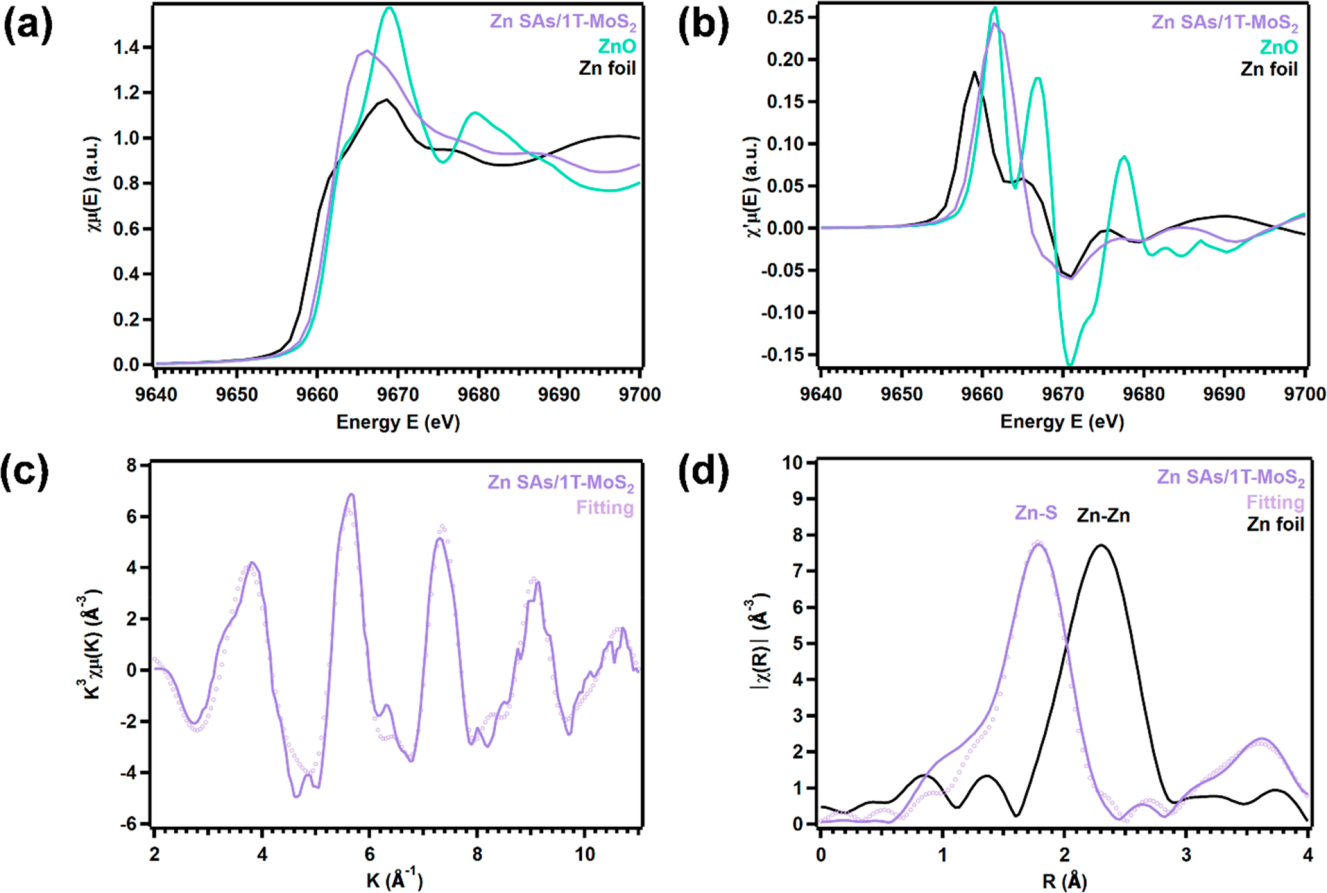
Zn K-edge XAS Characterization of Zn SAs/1T-MoS_2_. (a) XANES spectra with Zn foil and ZnO as reference samples. (b) Derivative of the XANES spectra. (c) EXAFS spectra in K space. (d) EXAFS spectra in R space.

To confirm the chemical states and atomic dispersion of the Zn SAs, the electronic and coordination structures of Zn SAs/1T-MoS_2_ were studied by X-ray absorption spectroscopy (XAS) at both the Zn K-edge (Figure 3) and the Mo K-edge (Figure S9). The X-ray absorption near edge structure (XANES) spectra (Figure 3a) show that the white line intensity and absorption edge of Zn SAs/1T-MoS_2_ are closer to ZnO than those of Zn foil, indicating that the SAs exist in the Zn^2+^ oxidation state. This is also reflected by the first derivative of the XANES spectra (Figure 3b). The *k*^3^-weighted extended X-ray absorption fine structure (EXAFS) spectra (Figure 3c) and Fourier-transformed EXAFS spectra in R-space (Figure 3d) correlate well with their best fitting lines modeled by DFT (Figure 5b), respectively, and suggest that Zn exists as SAs tetrahedrally coordinated to 4 S atoms. The coordination environment adopted by Zn SAs under confinement was first modeled as 1T-MoS_2_ layers confining a Zn SA that is octahedrally coordinated to the S basal planes. After structural optimization, the SA’s coordination geometry reorganized into a tetrahedral coordination with the S basal planes. Specifically, the Zn SAs formed asymmetric Zn–S1 and Zn–S3 coordination structures with the upper and lower S layers with bond lengths equal to 2.31 Å (Table S4). Typical peaks correspond to Zn–Zn bond formation (>2.50 Å) were not observed (Figure 3d), which indicates that the SAs remain atomically dispersed when under confinement.^59,60^ This tetrahedral coordination enables the Zn SAs to stabilize the expanded interlayer spacing of 1T-MoS_2_, which in turn, retains greater exposure of basal plane S active sites for enhanced HER performance.

Figure S9a shows the XANES spectra of Mo foil, 1T-MoS_2_, and Zn SAs/1T-MoS_2_ at the Mo K-edge corresponding to the 1s–5p transition. The edge energy of Zn SAs/1T-MoS_2_ is visibly higher than Mo foil and very close to 1T-MoS_2_, suggesting that the Mo atoms in Zn SAs/1T-MoS_2_ have an oxidation state similar to that of 1T-MoS_2_. This conclusion is further verified by the first derivative spectra of XANES (Figure S9b), where the valency of the Mo atoms in Zn SAs/1T-MoS_2_ is nearly the same as that of 1T-MoS_2_. The Mo K-edge EXAFS spectra in K-space (Figure S9c) and Fourier-transformed EXAFS spectra in R-space (Figure S9d) coincide well with the fitting line (also based on the DFT model in Figure 5b), indicating that the Mo atoms in Zn SAs/1T-MoS_2_ are octahedrally coordinated to 6 S atoms and an increase in S vacancies is not observed. The fitting results of the Fourier-transformed EXAFS spectra in R-space (Table S5) show that while half of the Mo–S bonds are at lengths that are expected for 1T-MoS_2_ (2.41 Å), the other half exhibit longer bond lengths equal to 2.62 Å (Table S5). This increase in the bond length may be ascribed to the slight distortion of octahedrally coordinated Mo–S centers induced by the intercalation of Zn SAs.

Altogether, these results confirm a few key findings. First, the Zn SAs adsorb between layers of 1T-MoS_2_ and are stabilized by Zn–S bonding interactions. Second, intercalating Zn SAs between layers of 1T-MoS_2_ expands the interlayer spacing by ∼3.4%. Third, the incorporation of Zn SAs does not influence the electronic properties or concentration of S vacancies as compared to that of pristine 1T-MoS_2_. Instead, the Zn SAs retain their 2+ oxidation state and d^10^ electronic configuration, which inhibits their ability to perform as active sites for the HER. Lastly, the Zn SAs tetrahedrally coordinate to basal S atoms and induce slight distortion of up to half of the Mo–S bonds in 1T-MoS_2_. Based on these findings, the impact of Zn confinement on HER catalysis in 1T-MoS_2_ is believed to solely be caused by confinement effects.

### HER Activity of Zn SAs/1T-MoS_2_

**Figure 4:**
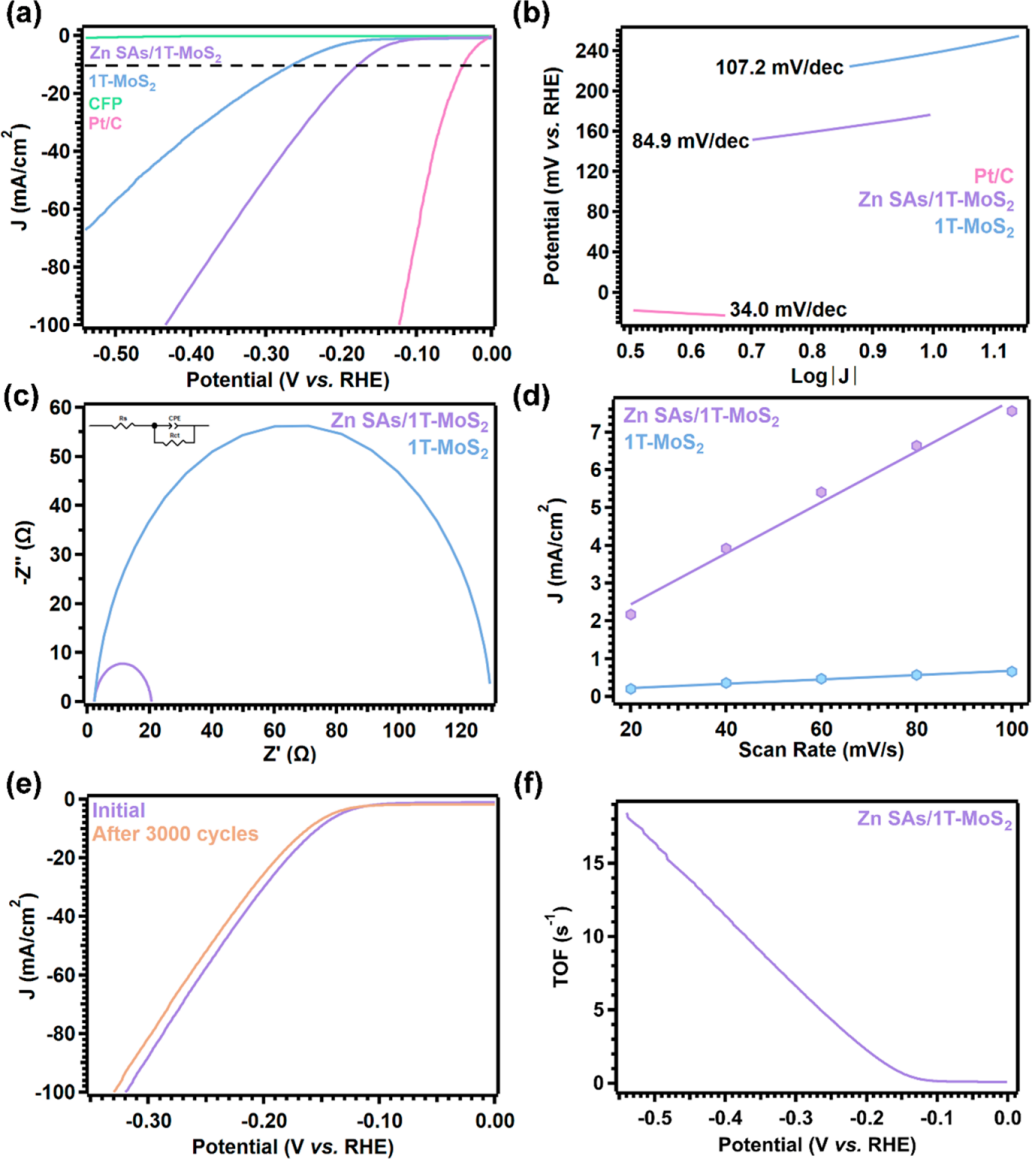
Electrochemical characterization of Zn SAs/1T-MoS_2_ in N_2_-saturated 0.5 M H_2_SO_4_ within a three-electrode configuration. (a) Polarization curves of HER for CFP, 1T-MoS_2_, Zn SAs/1T-MoS_2_, and Pt/C (20%). (b) Tafel plots of 1T-MoS_2_, Zn SAs/1T-MoS_2_, and Pt/C (20%). (c) EIS results for 1T-MoS_2_ and Zn SAs/1T-MoS_2_ fitted to the equivalence circuit are shown. (d) Scan-rate dependence of the current density at *E* = −300 mV vs RHE. (e) Electrochemical stability test of Zn SAs/1T-MoS_2_. (f) TOF values of Zn SAs/1T-MoS_2_.

Changes in HER performance with intercalation of Zn SAs were monitored electrochemically under acidic conditions (N_2_-saturated 0.5 M H_2_SO_4_) within a three-electrode configuration (detailed in the Methods). Linear sweep voltammograms (LSVs) were collected to measure the amount of overpotential (measured at −10 mA/cm^2^) 1T-MoS_2_ requires to drive HER and elucidate how the overpotential changes when Zn SAs are confined near basal plane active sites in 1T-MoS_2_. Comparison of overpotentials yielded by 1T-MoS_2_ intercalated with 2.5, 8.5, and 16.5 mg of Zn SAs shows that the overpotential peaks at 177 mV vs RHE with 8.5 mg of Zn SAs intercalated into 1T-MoS_2_ (Figure S10a, Table S6). The same trend is observed when the electrochemical impedance spectra (EIS) of the samples are analyzed to evaluate charge transport limitations (Figure S10b). The lowest charge transfer resistance (18.41 Ω) is observed when 8.5 mg of Zn SAs is intercalated into 1T-MoS_2_ (Table S7). These results indicate that intercalating 8.5 mg of Zn SAs enhances 1T-MoS_2_’s catalytic performance, while quantities above or below this amount exhibit worse HER activity and slow charge transport efficiency. When 8.5 mg of Zn SAs is intercalated, 1T-MoS_2_’s overpotential was reduced by 88 mV (Figure 4a, Table S6), indicating that less energy is required to drive HER when Zn SAs are spatially confined within 1T-MoS_2_’s interlayer spacing (Figure 4a).

Tafel slopes were extracted from the onset potential region of the LSVs to identify how the presence of Zn SAs changes the rate at which HER proceeds on 1T-MoS_2_ in acidic media (Figure 4b). In the first step of the HER mechanism, known as the Volmer step, a proton is reduced at an active site and adsorbed on the catalyst’s surface. In the second step, H_2_ is released through one of two pathways: either by a second proton/electron transfer, known as the Heyrovsky step, or the recombination of two adsorbed protons, known as the Tafel step.^61^ Since catalysts that exhibit the best HER performance yield the lowest overpotentials, these catalysts will produce the lowest Tafel slopes.^8^ Comparison of Tafel slopes produced by 1T-MoS_2_ (107.2 mV/dec) and Zn SAs/1T-MoS_2_ (84.9 mV/dec) shows a decrease of 22.3 mV/dec when Zn SAs are intercalated between 1T-MoS_2_ layers (Figure 4b). Spatial confinement has been predicted to increase the electrochemical potential of confined reacting species, which in turn, enhances charge transfer between the catalyst surface and adsorbed protons. As a result, less applied voltage is needed to produce the same current density.^20^ Here, the lowered kinetic barrier through which the HER mechanism proceeds is evidenced by the decrease in the Tafel slope observed when Zn SAs are under confinement. Comparison of these results to similar cases of SACs reported in literature reveals a similar trend in overpotential and Tafel slopes for 2H and 1T MoS_2_ supports intercalated with first-row transition metals that exhibit similar electronic configurations to Zn^2+^ (Table S8).

Confinement-induced changes in the charge transfer properties were evaluated by EIS (Figure 4c). A reduction in charge transfer resistance when Zn SAs are spatially confined within 1T-MoS_2_ is evidenced by the observably smaller radius of the semicircle produced by Zn SAs/1T-MoS_2_ compared to the radius of the semicircle produced by 1T-MoS_2_. Numerically, Zn confinement reduces charge transfer resistance in 1T-MoS_2_ from 127.5 to 18.41 Ω. This decrease in charge transfer resistance implies that the rate of charge transfer from the active sites to the adsorbed protons is increased with interlayer confinement of Zn SAs.

In catalysis, the rate of an electrocatalytic reaction is directly proportional to the active surface area. Therefore, cyclic voltammetry (CV, Figure S11) was employed to derive the double-layer capacitance and calculate the electrochemically active surface area (ECSA) of 1T-MoS_2_ and Zn SAs/1T-MoS_2_ (Figure 4d). Compared to the ECSA 1T-MoS_2_ yields (7 mF/cm^2^), Zn SAs/1T-MoS_2_ yields an ECSA equal to 29 mF/cm^2^, roughly four times greater than 1T-MoS_2_’s ECSA. As a measure of the catalyst’s surface area that is accessible to the electrolyte, this improvement implies that there is roughly four times more area available to facilitate charge transfer when Zn SAs are confined near basal plane active sites. This result corresponds well with the EIS results and may be correlated to the increase in fringe spacing induced by the Zn SAs in 1T-MoS_2_ that was observed in the HRTEM and XRD results (Figure 2).

To test the stability of Zn SAs/1T-MoS_2_ during catalysis, LSVs were collected both before and after the catalyst was subjected to 3,000 CV scans (Figure 4e). After 3,000 cycles, the overpotential increased by only 14 mV at 10 mA/cm^2^. The excellent stability was further evidenced by continuous electrolysis at −0.2 V vs RHE which showed nearly unchanged current generation after 24 h (∼20 mA cm^–2^, Figure S12). XPS spectra of Zn SAs/1T-MoS_2_ on carbon fiber paper (CFP) were collected before and after collecting 3,000 CV scans to evaluate the catalyst’s structural stability (Figure S13, Table S9). The presence of both MoS_2_ (Mo^4+^) and MoS_*x*_ (Mo^5/6+^) is confirmed by the pre- (Figures S13a,c,e,g) and post-CV (Figures S13b,d,f,h) XPS spectra. While Zn^2+^ SAs are observed near the catalyst surface before running CVs (Figure S13a), they are lost after the CV scans are completed (Figure S13b). Prior to stability testing, small amounts of MoO_3_ (Figures S13c,g) and sulfate (SO_4_^2–^, Figures S13e,g) are observed on the sample surface. After stability testing, MoO_3_ disappears (Figures S13d,h) and the layer of SO_4_^2–^ present on the sample grows (Figures S13f,h). Specifically, the sample initially starts with 4.55 at% of SO_4_^2–^. After CV is completed, 20.56 at% of SO_4_^2–^ is observed on the sample’s surface. Furthermore, the Zn 2p peaks reappear after the sample is sputtered for 30 s (Figure S14). Based on these results, the following observations were made. O_2_ has been reported in the literature to form Mo-oxide species by bonding to unsaturated Mo atoms at vacancy sites on defect-rich MoS_2_ surfaces via chemical adsorption.^62,63^ Furthermore, O_2_ has been reported to not alter the electronic properties of MoS_2_.^63^ Therefore, the complete loss of MoO_3_ after electrolysis indicates that this species is localized along the sample surface and is likely caused by surface oxidation during working electrode preparation. Meanwhile, interactions between the electrolyte and catalyst surface during HER drive the growth of SO_4_^2–^ on the catalyst surface. The significant increase of SO_4_^2–^ on the catalyst surface likely inhibits the ability to see the Zn 2p peaks after electrolysis, considering that the concentration of intercalated Zn SAs is very low (1.0% as determined by ICP-OES, Table S10). Since SO_4_^2–^ is a soluble ion, once the sample is submerged into the electrolyte, SO_4_^2–^ may redissolve. The reappearance of Zn 2p peaks after sputtering the sample for 30 s indicates that the Zn SAs intercalated between layers beneath the catalyst surface remain intact. The lack of significant decline (14 mV shift) in HER performance after 3,000 CVs observed further confirms that the integrity of the catalyst structure is well maintained (Figure 4e).

As previously mentioned, an average mass percent of 1.0% Zn SAs in Zn SAs/1T-MoS_2_ as quantified by ICP-OES (Table S10) was employed to determine turnover frequencies (TOF) and evaluate the catalyst’s efficiency toward HER (Figure 4f). Similar to the polarization curves, the TOF values increase with higher potential. This result aligns well with reports from literature as well.^64^ In our previous work, 1T-MoS_2_ substituted with Ni SAs yielded a TOF of 0.7 s^–1^ at 130 mV overpotential.^37^ Here, the intercalation of Zn SAs between layers of 1T-MoS_2_ yields a TOF equal to 1.40 s^–1^ at 177 mV overpotential when the current density is 10 mA/cm^2^. Overall, the effects of Zn confinement within 1T-MoS_2_ layers include decreased overpotentials, lowered kinetic barriers_,_ faster charge transfer rates, and increased active surface area, all while retaining excellent stability.

### Correlation of Confinement Effects to HER activity

First-principles DFT calculations were performed to elucidate how confined Zn SAs influence 1T-MoS_2_’s catalytic performance. Initially, two positions occupied by a Zn SA located above the basal plane of a single layer of 1T-MoS_2_ were considered (Figure S15). In the first structure, the Zn SA was positioned above a Mo atom (single layer Zn SAs/1T-MoS_2_, Model I). In the second structure, the Zn SA is positioned above an S atom (single layer Zn SAs/1T-MoS_2_, Model II). Compared to the normal length of Zn–S bonds (∼2.36 Å), both coordination structures yield elongated Zn–S bond lengths that are greater than 2.95 Å.^65^ Therefore, the formation of Zn SAs/1T-MoS_2_ is predicted to be unfavorable for Models I and II with only a single layer of 1T-MoS_2_. Instead, two or more layers of 1T-MoS_2_ are required to stabilize intercalated Zn SAs and properly investigate the catalytic effects of spatially confining Zn SAs within the microenvironment of the 1T-MoS_2_ active sites.

In an effort to understand why the Zn SAs prefer to adsorb to the basal plane on top of Mo atomic positions instead of substituting within the 1T-MoS_2_ lattice, local active configurations demonstrating the possible atomic positions of Zn SAs on 1T-MoS_2_ were constructed and their resulting formation energies for each configuration were compared. In total, there are four possible anchoring sites for Zn SAs on 1T-MoS_2_ (Figure S16). The reported chemical potentials used to calculate the formation energies discussed herein for S, Mo, and Zn were taken from an S8 molecule, Mo metal, and Zn metal, respectively. In the first model (Figure S16a), a Zn SA was tetrahedrally coordinated to 1T-MoS_2_ with one bond formed with the top layer’s basal plane and three bonds formed with the bottom layer’s basal plane, respectively. In this configuration, the Zn atom is adsorbed on top of an Mo atom, and the formation energy is −6.06 eV. In the second model (Figure S16b), a Zn SA was tetrahedrally coordinated to form three bonds with the upper basal plane and one bond with the lower basal plane. However, the instability of this configuration caused the model to rearrange itself into a tetrahedral coordination that forms two bonds with the upper basal plane and two bonds with the lower basal plane. In this configuration, the Zn atom adsorbs on top of an S atom and the formation energy is −5.77 eV. In the third configuration (Figure S16c), an Mo atom is substituted by a Zn atom, which generates a formation energy equal to −2.14 eV. In the fourth configuration (Figure S16d), a Zn atom substitutes an S atom in the lower basal plane, which generates a formation energy equal to −4.16 eV. Comparison between the different formation energies of the four configurations shows that the first configuration with the most negative formation energy (Figure S16a, −6.06 eV) is the most likely configuration to occur due to being the most thermodynamically stable configuration. This result corresponds with the experimental STEM results (Figure 1).

**Figure 5:**
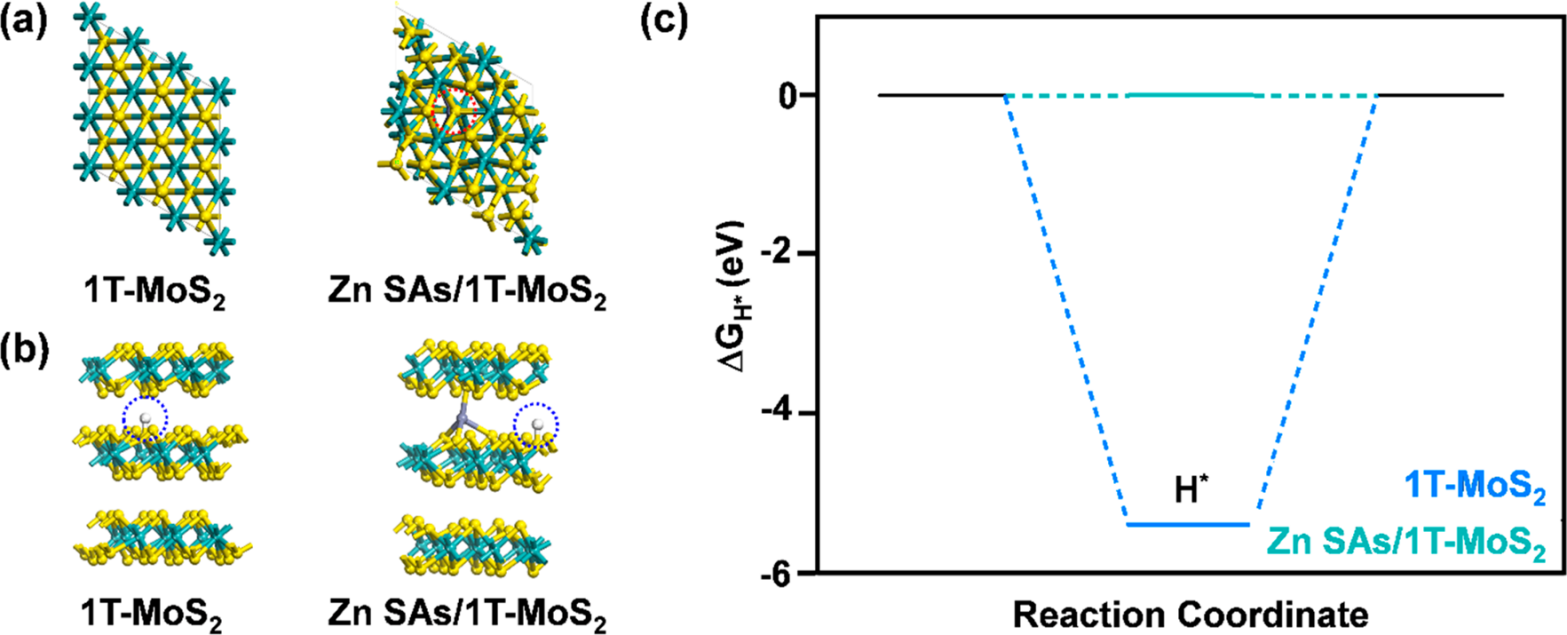
DFT computational analysis of 1T-MoS_2_ and Zn SAs/1T-MoS_2_. (a) Top view. The Zn SA adsorption site between the 1T-MoS_2_ layers is circled in red. (b) Side view. Optimal adsorption modes of H* adsorption sites are circled in blue. (c) Calculated Δ*G*_H*_ for HER at 0 V vs RHE and pH = 0. The yellow, cyan, purple, and white spheres represent S, Mo, Zn, and H atoms, respectively.

To understand how confining Zn SAs within the microenvironment of 1T-MoS_2_ active sites changes the proton adsorption/desorption kinetics of 1T-MoS_2_, Δ*G*_H*_ values for a trilayer of 1T-MoS_2_ and a trilayer 1T-MoS_2_ intercalated with a tetrahedrally coordinated Zn SA were calculated for HER at a potential of 0 V vs RHE and pH = 0 (Figure 5). Predicted models of the top view and optimal adsorption modes of H* for both cases are demonstrated in Figures 5a, 5b. The extremely negative Δ*G*_H*_ value (−5.40 eV) yielded by trilayer 1T-MoS_2_ indicates that the adsorption of protons to the S active sites will be strong. Consequently, the desorption of adsorbed protons upon producing H_2_ will be challenging, and sluggish reaction kinetics are expected for active sites located along the basal plane. In contrast, Zn SAs/1T-MoS_2_ yields a much more thermoneutral Δ*G*_H*_ (0.00294 eV), indicating that HER adsorption and desorption kinetics will be much more facile when Zn SAs are confined near the basal plane active sites in 1T-MoS_2_. Altogether, these calculations correspond well with the experimentally observed improvement in the HER performance.

Mulliken charge analysis was employed to predict the changes in the electron density of S active sites along the basal plane when Zn SAs are confined within their microenvironments (Figure S17). Here, 3D isosurface diagrams of the differential charge are displayed for Zn SAs/1T-MoS_2_. Charge clouds shown in blue represent energetically negative regions where the atom has lost electrons. Charge clouds shown in red exhibit positive charge and represent atoms that have gained electrons. The tetrahedral coordination of Zn SAs to basal S atoms yields a thermoneutral free energy value, making this coordination structure ideal for fast HER kinetics. In this coordination structure, the Zn atom donates electrons to nearby basal plane S atoms on 1T-MoS_2_, resulting in the formation of strong ZnS bonds. This electron transfer changes the adsorption behavior of H atoms attached to neighboring S active sites. Based on these predictions, one may deduce that confining Zn SAs enhances the catalytic activity of 1T-MoS_2_ by increasing the electron density surrounding the S active sites around SAs. As a result, the catalytic activity of 1T-MoS_2_ is enhanced accordingly.

To reveal the influence of Zn intercalation on the band structure of 1T-MoS_2_, the PDOS spectra of the Zn 4s (black), Zn 3d (blue), S 3s (green), and S 3p (purple) orbitals were evaluated (Figure S18). Results show a strong electronic overlap between the Zn 4s and S 3p orbitals. Together with the Mulliken charge population analysis of the atomic orbitals (Figure S17), one may deduce that the electrons transfer from the Zn 4s orbital to the S 3p orbital during Zn–S bond formation, which in turn causes the Zn atom to lose electrons and exhibit a positive valence state. Consequently, the S atoms coordinated to the adsorbed Zn atom gain electrons and exhibit a more negative valence state than they would in bare 1T-MoS_2_. These results match well with the experimental XPS results reported herein (Figure S5). In regard to the effects of electronic accumulation at S sites on H* adsorption, near the Fermi level the density of states mainly comes from S atoms, while the contribution from Zn is close to 0 eV. Therefore, the Zn atoms exhibit weak adsorption capacity for H* and are thus inactive, which is consistent with the H* adsorption configuration. In contrast, the S atoms have available p orbitals that can form bonds with H*. The observed electron transfer from Zn to neighboring S atoms (Figures S17 and S18) leads to an increase in electron filling and decrease in empty orbitals near the Fermi level, which weakens the electron accepting ability of S sites and H* adsorption.

To further elucidate the influence of Zn intercalation on the electronic structure and resulting HER activity of 1T-MoS_2_, the PDOS spectra of the s and p orbitals in 1T-MoS_2_ and Zn SAs/1T-MoS_2_ were compared (Figure S19). As elucidated from Figure S18, electron transfer from Zn to neighboring S atoms results in a higher extent of electron filling of neighboring S atoms. As shown by Figure S19a, this leads to a negative shift in the p band center of S atoms in Zn SAs/1T-MoS_2_ from −3.78 eV (red dashed line) to −4.0 eV (green dashed line) and a decrease in the intensity of state density compared to 1T-MoS_2_ near the Fermi level (shown in the black dashed box). Since the electronegativity of the S atom is stronger than a proton’s electronegativity, the protons will donate electrons to active S sites in the adsorption process. However, the increase in electronic occupation of the S 3p orbitals and decrease in empty orbitals near the Fermi level caused by Zn intercalation will cause a reduction in the electron accepting ability of the S atoms. Although the adsorption of protons is not strictly linear with the atomic electron density of S, it is negatively correlated. Thus, the number of electrons transferred to 1T-MoS_2_ induced by proton adsorption is reduced after Zn intercalation. Proton adsorption capacity reduces from −5.40 to 0.5819 eV after Zn intercalation, which in turn sharply increases the catalyst’s HER activity.

Since Zn intercalation influences the electronic structure of the catalyst’s entire surface, we also calculated the H* adsorption behavior of some typical sites (Figure S20). The results suggest that Zn intercalation causes the HER activity of the catalyst’s entire surface to improve, as shown by the resulting Δ*G*_H*_ values being closer to 0 eV for all of the H* adsorption sites considered. Meanwhile, compared with the neighboring S atoms in the first coordination sphere, the adsorption capacity of neighboring S atoms in the second coordination sphere is also weakened and yields better HER activity than the S atoms in the first coordination sphere (0.00294 eV).

To explore the influence of interlayer spacing expansion on the HER activity of 1T-MoS_2_, we manually set the lattice parameters of the supercell to increase by 3.4% along the Z axis before structural optimization (Figure S21a). By fixing the lattice parameters, only the structure of the ion step can be changed during structural optimization (Figure S21b). Calculation results show a Δ*G*_H*_ of −4.8081 eV, far worse than that of Zn SAs/1T-MoS_2_ (0.00294 eV). In this regard, the electronic regulation of neighboring S atoms is believed to be the root cause of the observed improvement in HER activity upon intercalation of Zn SAs between layers of 1T-MoS_2_. By comparison, the lattice spacing expansion is only a secondary factor.

## Conclusions

In this work, the catalytic effects of confining Zn SAs within the interlayer spacing of 1T-MoS_2_ were investigated, and several key findings were revealed. First, the Zn SAs adsorb to the basal plane of 1T-MoS_2_. Second, this adsorption behavior allows the Zn intercalants to expand the interlayer spacing of 1T-MoS_2_ by ∼3.4%. Third, as SAs exist in the Zn^2+^ oxidation state, the Zn intercalants tetrahedrally coordinate to nearby S atoms and donate electrons to basal S atoms. While they do induce a slight distortion in the 1T-MoS_2_ lattice, the octahedral coordination surrounding the Mo atoms remains unchanged. Zn confinement does not alter the amount of S vacancies or magnetic properties significantly. Catalytically, spatial confinement of Zn SAs within 1T-MoS_2_ causes an 88 mV decrease in overpotential, 22.3 mV/dec decrease in Tafel slope, 109.09 Ω drop in charge transfer resistance, and retains excellent stability. Based on the in-depth structural analysis reported herein, the significant improvement in catalytic performance observed is attributed to the effects of confining Zn SAs within the microenvironments of basal plane active sites in 1T-MoS_2_. DFT and PDOS calculations predict Zn SAs/1T-MoS_2_ to yield a much more thermoneutral Δ*G*_H*_ (0.00294 eV) compared to that of 1T-MoS_2_ (−5.40 eV), suggesting that HER adsorption and desorption kinetics will be much more facile when Zn SAs are under confinement.

This work reveals that intercalating transition metal ions such as Zn^2+^ into catalytic layered materials such as 1T-MoS_2_ enables the electronic states of confined microenvironments to be controlled according to the guest metal’s coordination geometry and electronic states. In turn, the 2D support’s catalytic activity may be enhanced accordingly. Although this work investigates the confinement of Zn SAs within the microenvironments of 1T-MoS_2_’s basal plane active sites and its influence on HER performance, the basis of this work may easily be adopted to other types of catalytic reactions and layered materials. Since confinement effects have been recognized for their importance in heterogeneous, homogeneous, and enzymatic catalysis, the knowledge gained from this work may be appropriately applied to all fields of catalysis.

## Methods

### Chemicals

Ammonium heptamolybdate tetrahydrate [(NH_4_)_6_Mo_7_O_24_·6H_2_O, 99% Alfa Aesar] and thioacetamide (CH_3_CSNH_2_, 99+% Acros Organics) were used without further purification.

### Synthesis of 1T-MoS_2_ Nanosheets

First, 50 mg of (NH_4_)_6_Mo_7_O_24_·6H_2_O, 80 mg of CH_3_CSNH_2_, and 10 mL of deionized (DI) water were combined in a 25 mL autoclave and sonicated until they were fully dissolved. Once dissolved, the autoclave was sealed within a hydrothermal reactor and heated at 180°C for 24 h. Once the reactor cooled to room temperature, the solution of crude product was transferred to 15 mL conical centrifuge tubes. The crude product was washed and centrifuged three times: once with DI water, once with ethyl alcohol, and once with acetone. The solutions were centrifuged for 10 min at ∼4,000 rpm in between each washing step and the supernatant layer was removed after each centrifugation step. After washing, the purified product was dried in a vacuum oven at ∼80 °C and ∼25 mmHg for 24 h. The dried and purified product was finely ground with a mortar and pestle and stored under ambient conditions.

### Synthesis of Zn SAs/1T-MoS_2_

First, 50 mg of 1T-MoS_2_, 15 mL of DI water, and 35 mL of ethanol were combined and continuously stirred at room temperature during the entire synthesis. Next, 2.5, 8.5, and 16.5 mg of ZnCl_2_ was dissolved in 6 mL of DI water to prepare Zn SAs (2.5 mg)/1T-MoS_2_, Zn SAs (8.5 mg)/1T-MoS_2_, and Zn SAs (16.5 mg)/1T-MoS_2_, respectively. A syringe pump apparatus was employed to inject the Zn solution into the 1T-MoS_2_ solution. To do this, the entire solution of ZnCl_2_ was injected into the 1T-MoS_2_ solution at a flow rate of 10 μL/min flow rate. The solution stirred continuously at room temperature for 24 h after the injection was complete. Afterward, the solution was transferred to 15 mL Conical Centrifuge Tubes, centrifuged for 10 min at ∼4,000 rpm, washed with DI water, and centrifuged again for the same amount of time. The supernatant layer was removed after each centrifugation step. The purified product was dried in a vacuum oven at ∼80°C and ∼25 inHg for 24 h, then finely ground with a mortar and pestle and stored under ambient conditions.

### Characterization

XRD patterns were measured using a Panalytical X’Pert multipurpose XRD with Cu–Kα radiation (λ = 1.5418 Å) at 45 kV and 40 mA, with step size = 0.02 and scan step time = 17.75 s. The measurement range was from 10° to 80° in terms of 2θ. XPS measurements were collected with a PHI 5600 XPS system equipped with a monochromatic Al Kα X-ray source and Omni Focus III lens operating at 250 W, 14 kV, 600 μm^2^ spot size, and a maximum base pressure of 5 × 10^–9^ Torr. A 90° angle was maintained between the X-ray source and analyzer. Survey spectra were collected with 117.4 eV pass energy, 1.0 eV/step, and 50 μs dwell time. Multiplexes were collected using 11.75 eV pass energy, 0.050 eV/step, and 50 μs dwell time. The instrument was calibrated to Au 4f_7/2_ = 84.00 eV and Cu 2p_3/2_ = 932.67 eV immediately prior to collecting the data. All spectra were calibrated to C 1s = 284.8 eV.^66^ Multiplexes were fitted by using IgorPro XPS Tools Software. A Shirley background and 80% Lorentzian–Gaussian were employed for all peak analyses. FTIR spectra were collected using a PERKIN ELMER CE-440. Raman spectra were recorded using a Thermo Scientific DXR Raman Spectrometer employing an Ar-ion laser operating at 532 nm, a 50 μm pinhole, and 3.0 mW laser power. EPR characterization was carried out on a Bruker EMX spectrometer (X-band) operating at a frequency of ∼9.46 GHz. Field frequency modulation, modulation amplitude, and microwave power were set to 100 kHz, 0.4 mT, and 2.0 mW, respectively, in every case to avoid saturation effects. All EPR measurements were recorded at room temperature.

Aberration-corrected scanning transmission electron microscopy (AC-STEM) was performed using the JEOL Grand ARM equipped with two spherical aberration correctors at 300 kV. High-angle angular dark-field (HAADF) STEM images were acquired by a convergence semiangle of 22 mrad and inner and outer collection angles of 83 and 165 mrad, respectively. Energy dispersive X-ray spectroscopy (EDX) was conducted by using JEOL dual EDX detectors and a specific high count analytical TEM holder. Sample compositions were analyzed by a PerkinElmer Optima 3000 DV ICP-OES. Commercially available Copper standard solutions (1000 mg L^–1^ in nitric acid, Sigma-Aldrich) were used for calibration. The standards were diluted to 1000 ppb (ng g^–1^), 500, 100, 50, and 1 ppb, respectively, by mixing acid solutions (5 v/v% HCl + 5 v/v% HNO_3_) to establish the calibration curves. All samples were dissolved in concentrated HNO_3_ and then diluted to a concentration of 5% with DI water. Zn SAs/1T-MoS_2_ was diluted 100 times using 5% HNO_3_ for measurements.

Mo K-edge XAS spectra were collected at beamline 4–1 from the Stanford Synchrotron Radiation Lightsource (SSRL). The X-ray fluorescence was detected by a Lytle-type fluorescence-yielding ion chamber detector. To reduce background noise from elastic scattering, the Soller slits were aligned and fitted with suitable Z-1 filters. Mo K-edge XAS data was measured within the range 19.778–20.887 keV in fluorescence mode with a step size of 0.25 eV at the near edge. The Zn K-edge XAS was run within the 9.46–10.50 keV range in fluorescence mode with a step size of 0.25 eV at the near edge. All samples were prepared by placing a small amount of homogenized powder mixed with boron nitride (via agate mortar and pestle) on 3 M Kapton Polyimide tape, which was purchased from 3M (https://www.3m.com/).

### Electrochemical Measurements

All electrochemical measurements were conducted in a N_2_-saturated 0.5 M H_2_SO_4_ electrolytic solution within a three-electrode configuration. A CHI 660E electrochemical workstation was used for all electrochemical measurements. Graphite was employed as the counter electrode and Ag/AgCl (3 M NaCl, BASI) as the reference electrode. Electrodes were prepared by drop casting 200 μL of catalyst ink (50 μL each time, repeated 4 times) onto a 1 × 2 cm^2^ piece of carbon fiber paper (CFP). The loading of the catalysts on CFP is 1 mg/cm^2^. All potentials reported were calibrated with respect to the Ag/AgCl reference electrode in acidic media (0.5 M H_2_SO_4_) using eq 1:
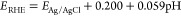
1LSVs were conducted under ambient conditions from 0 to −0.8 V with a 5 mV/s scan rate, 1 mV step size, and 0.001 A/V sensitivity. Onset potentials used to determine the Tafel slopes were extracted from the LSVs and defined as the potential at which the current began to increase (0.05 mA/cm^2^). Overpotentials were measured at −10 mA/cm^2^. EIS measurements were performed at −0.4 V with a 0.005 V variation in the frequency range of 1–10^5^ Hz and 12 steps per decade. The electrochemical active surface area (ECSA) was derived from cyclic voltammograms (CV) measured with varying scanning rates of 20, 40, 60, 80, and 100 mV s^–1^. The stability of Zn SAs/1T-MoS_2_ was evaluated by running CV for 3,000 cycles from 0 to −0.8 V, followed by comparison of the initial and final LSV curves. The turnover frequency (TOF) was reported as the TOF corresponding to the overpotential reported (177 mV vs RHE).

### Computational Methods

Density functional theory (DFT) calculations were performed by using CASTEP coding. The electronic exchange-correlation potential was conducted using the Perdew–Burke–Ernzerhof (PBE) functional of the generalized gradient approximation (GGA) and the ultrasoft pseudopotentials were used. The kinetic energy cutoff was set to 400 eV for the plane-wave basis set. Brillouin zone integration was sampled with the 3 × 3 × 1 MonkhorstPack mesh K-point for bulk and surface calculations, respectively. The DFT dispersion correction (DFT-D) method was used to correct for the van der Waals interactions. A three-layer repeating unit supercell with the formula Mo_27_S_54_ and Mo_27_S_54_Zn were constructed for bulk calculations. Monolayered 1T-MoS_2_ (Mo_9_S_18_), Zn SAs/1T-MoS_2_ (Mo_9_S_18_Zn), with a vacuum region of 15 Å along the Z axis, was constructed based on the HAADF-STEM imaging. The convergence tolerances were set to 1 × 10^–5^ eV per atom for energy, 1 × 10–3 Å for maximum displacement, and 0.03 eV Å^–1^ for maximum force. The thermodynamic energies and Gibbs free energies Δ*G*_H*_ were calculated using eq 2:
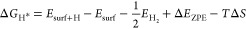
2where Δ*E*_ZPE_ and Δ*S* are the difference in the zero-point energy and entropy between the adsorbed H atom and the gaseous phase H_2_. At 300 K, Δ*G*_H*_ may be calculated using eq 3:
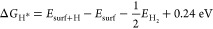
3

## Supporting Information Available

HAADF-STEM images, FTIR spectra, EDX mapping, ICP-OES analysis, Raman spectra and assignment, XPS spectra and assignment, EPR spectra and g-value assignments, XAS characterization and fitting parameters, electrochemical data and fitting, DFT models, and Mulliken charge analysis (PDF)
