# Supplementary material for: Zinc Single Atom Confinement Effects on Catalysis in 1T-Phase Molybdenum Disulfide
Source: ACS Nano. 2023 Jan 11;17(2):1414–26. doi: 10.1021/acsnano.2c09918 (PMC9878712; doi:10.1021/acsnano.2c09918)
Supplement: Supplementary file 1 — nn2c09918_si_001.pdf [file nn2c09918_si_001.pdf]

Supporting Information

Zinc Single Atom Confinement Effects on Catalysis in 1T-phase Molybdenum Disulfide

*Sabrina M. Younan[a],‡, Zhida Li[a,b],‡, XingXu Yan[c], Dong He[e], Wenhui Hu[f], Nino Demetrashvili[a], Gabriella Trulson[a], Audrey Washington[a], Xiangheng Xiao[e], Xiaoqing Pan[c,d], Jier Huang[f], Jing Gu[a],**

[a] Department of Chemistry and Biochemistry, San Diego State University, 5500 Campanile Drive San Diego, CA 92182, USA

[b] State Key Laboratory of Urban Water Resource and Environment, School of Civil and Environmental Engineering, Harbin Institute of Technology, Shenzhen 518055, China

[c] Department of Materials Science and Engineering, University of California, Irvine, Irvine, CA 92697, USA

[d] Department of Physics and Astronomy, University of California, Irvine, Irvine, CA 92697, USA

[e] Department of Physics, Wuhan University, Wuhan 430072, China

[f] Department of Chemistry, Marquette University, Milwaukee, WI 53201, USA

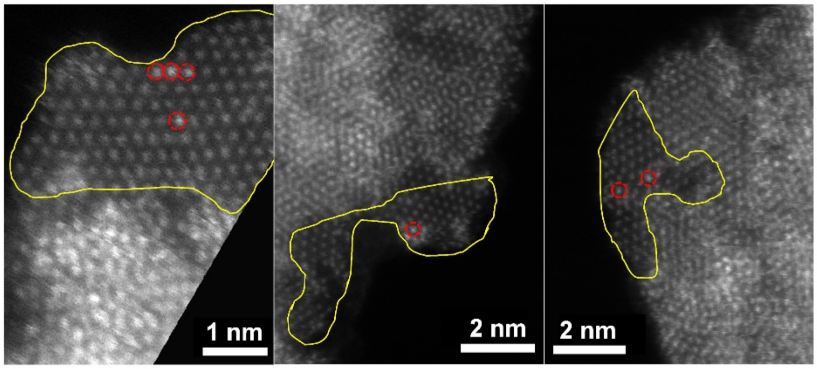

Figure S1. Identification of the adsorption model of Zn SAs/1T-MoS_2 by STEM. Monolayer regions of 1T-MoS_2 sheets are outlined in yellow. The red circles highlight brighter atomic positions where Zn atoms are located on top of Mo atoms.

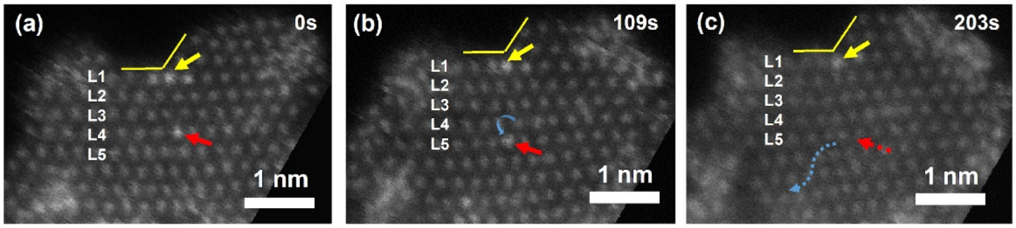

Figure S2. Migration of Zn SAs on 1T-MoS_2. STEM images of continued electron beam irradiation for: **(a)** 0 s, **(b)** 109 s, and **(c)** 203 s. Scale bars are all 1 nm. Yellow lines and arrows indicate the edge of this region, while the Zn SA acts as a reference to align the images. The Zn SA indicated by the red arrow moves from the L4 to L5 at 109 s, then disappears after 203 s. The maximum energy E_m transferred from a high energy electron with an accelerating voltage of E to the atomic nucleus may be calculated using the following equation:[1, 2]

$$E_{\mathrm{m}} = 2.1477 \times 10^{-9} \times E(E + 1.022 \times 10^{6}) / A$$

where A is the atomic mass and the energies are in unit of eV. For the 60-keV electron beam used in our experiments, the E_m for Zn atoms is 4.65 eV. Such energy is larger than the bonding

energy of Zn-S (1.06-2.63 eV).[3] Therefore, the electron beam is expected to be able to drive the migration of Zn SAs adsorbed along the 1T-MoS_2 basal plane.

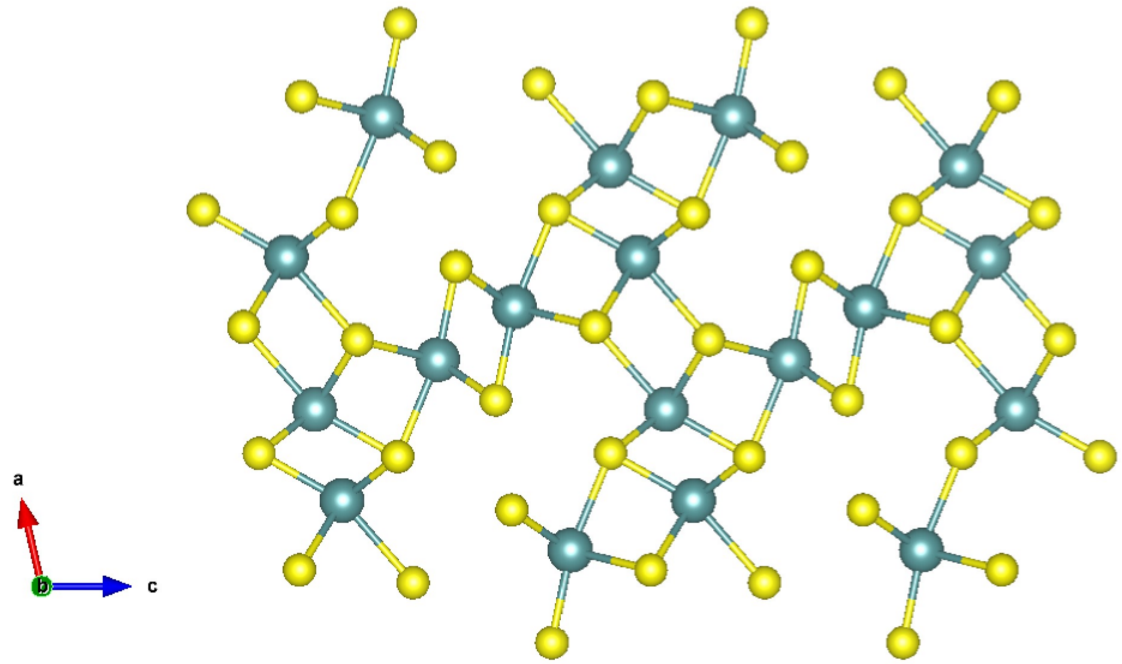

Figure S3. Lattice structure of monoclinic Mo_2S_3.

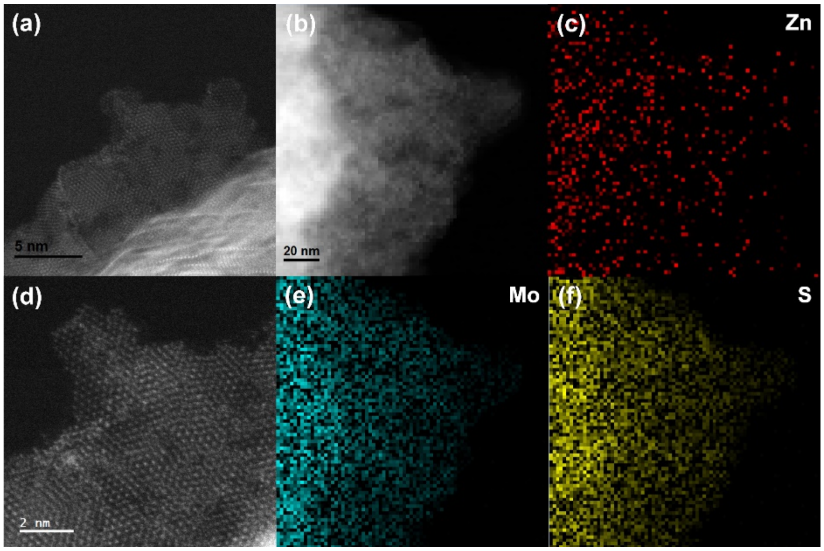

Figure S4. STEM and EDX mapping of Zn SAs/1T-MoS_2.

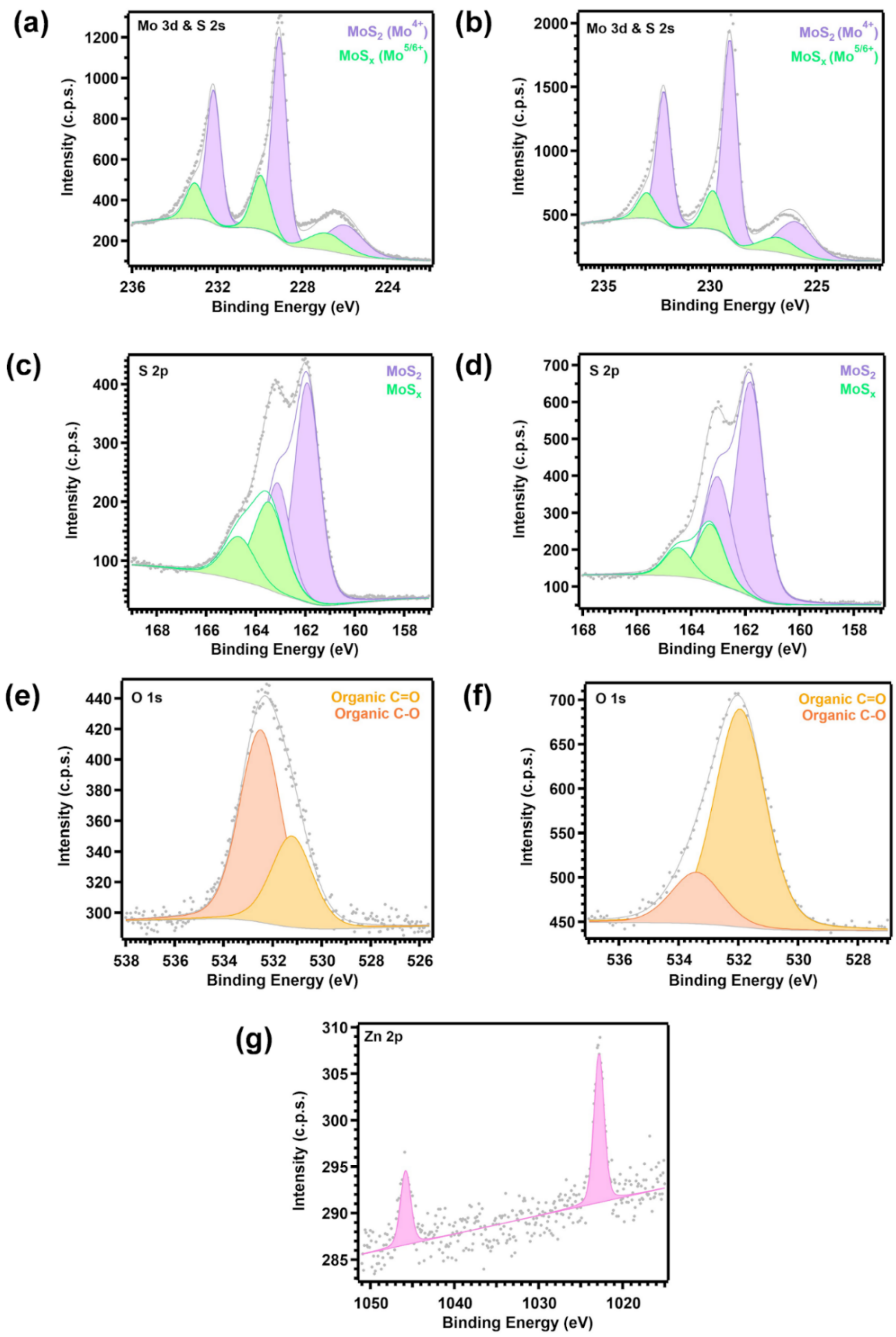

Figure S5. XPS characterization of 1T-MoS_2 (**a, c, e**) and Zn SAs/1T-MoS_2 (**b, d, f, g**). (**a-b**) Mo 3d and S 2s spectra. (**c-d**) S 2p spectra. (**e-f**) O 1s spectra. (**g**) Zn 2p spectrum.

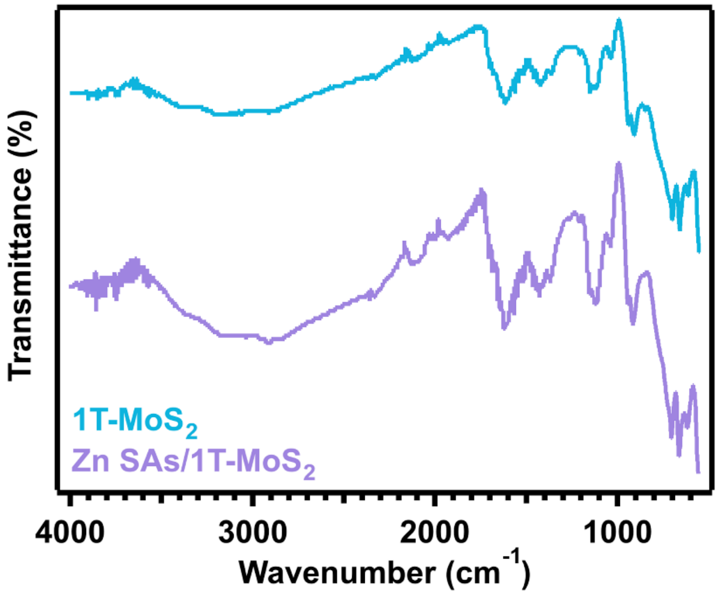

Figure S6. FTIR spectra of Zn SAs/1T-MoS_2 (purple) and 1T-MoS_2 (blue).

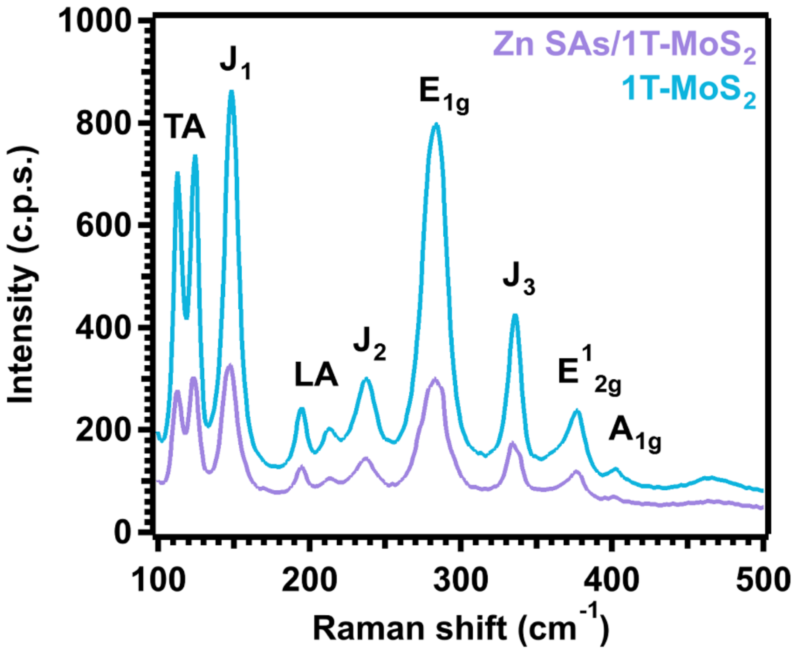

Figure S7. Raman spectra of Zn SAs/1T-MoS_2 (purple) and 1T-MoS_2 (blue).

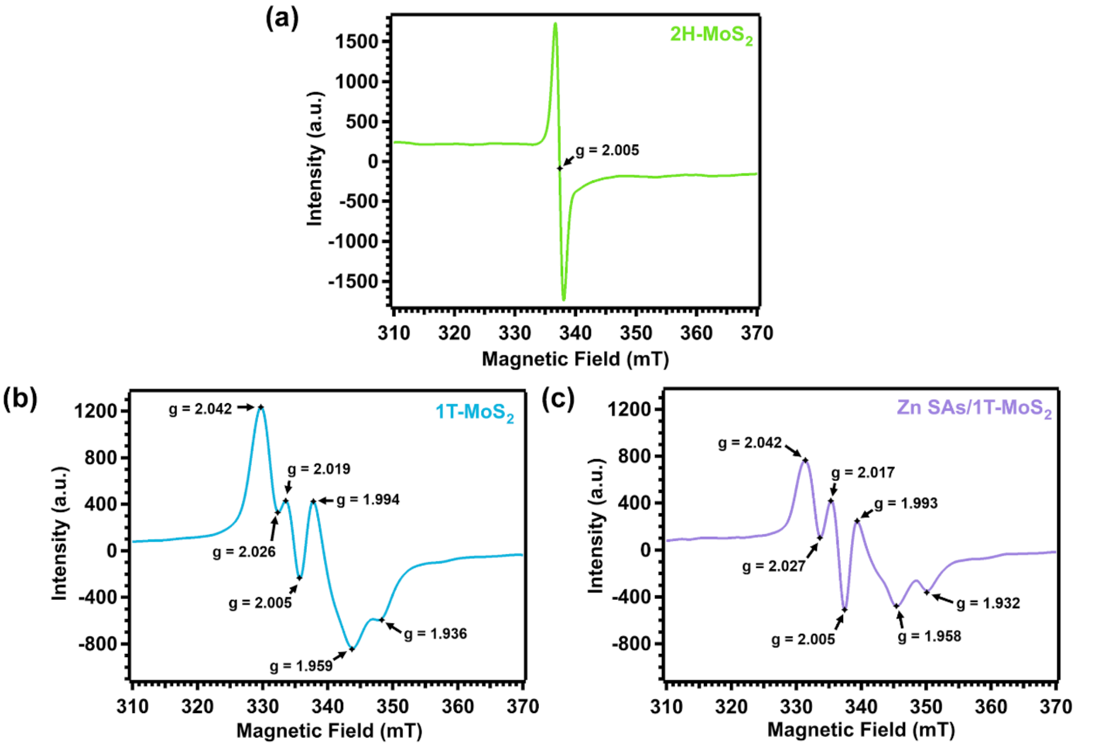

Figure S8. EPR spectra of (**a**) 2H-MoS_2, (**b**) 1T-MoS_2, and (**c**) Zn SAs/1T-MoS_2.

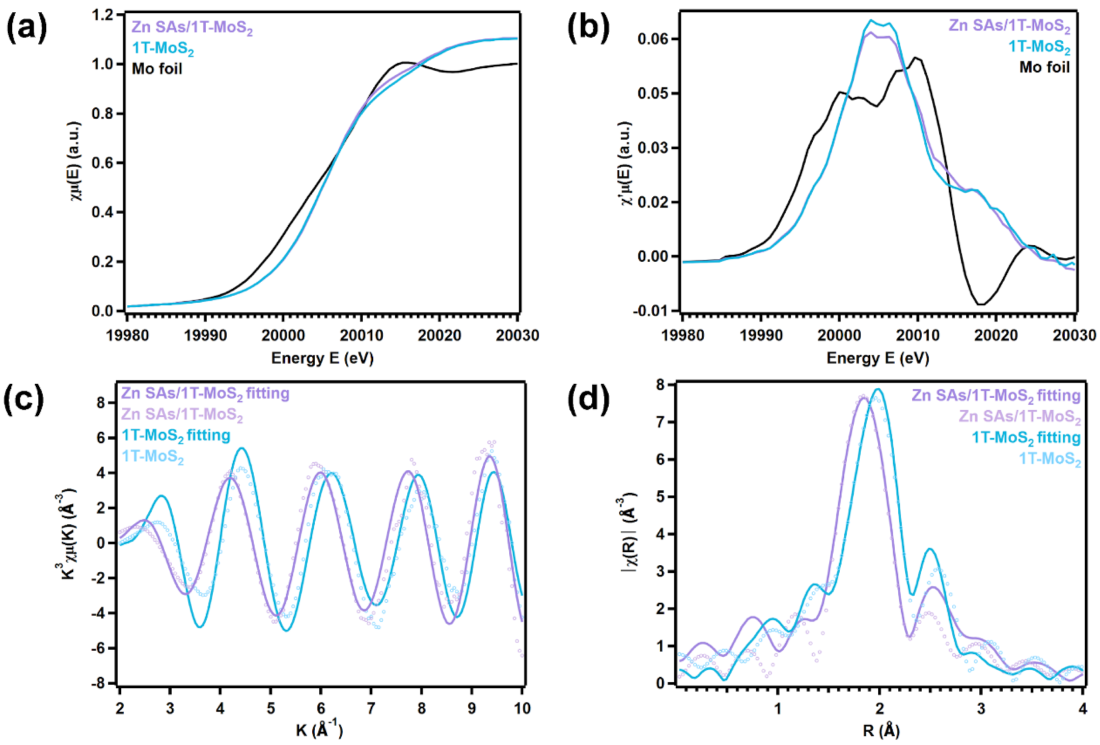

Figure S9. Mo K-edge XAS characterization of Zn SAs/1T-MoS_2. (**a**) XANES spectra with Mo foil and 1T-MoS_2 as reference samples. (**b**) Derivative of XANES spectra. (**c**) EXAFS spectra in k space. (**d**) Fourier-transformed EXAFS spectra in R space.

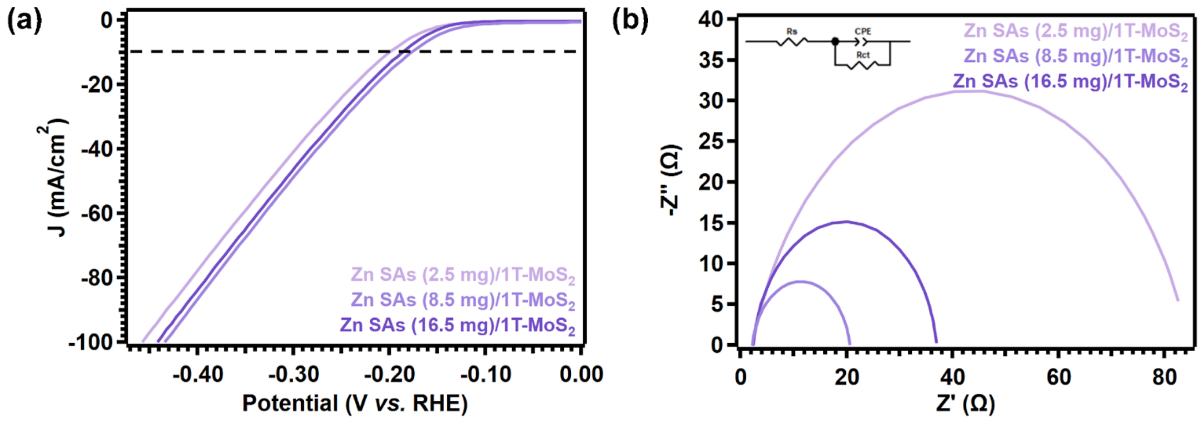

Figure S10. Electrochemical analysis of how the HER performance of 1T-MoS_2 changes as the quantity of Zn single atoms (SAs) increases. These measurements were performed within a standard three-electrode configuration, with N_2-saturated 0.5 M H_2SO_4 as the electrolyte. **(a)** Linear sweep voltammograms (LSVs). **(b)** Electrochemical impedance spectroscopy (EIS) fitted to the equivalent circuit shown.

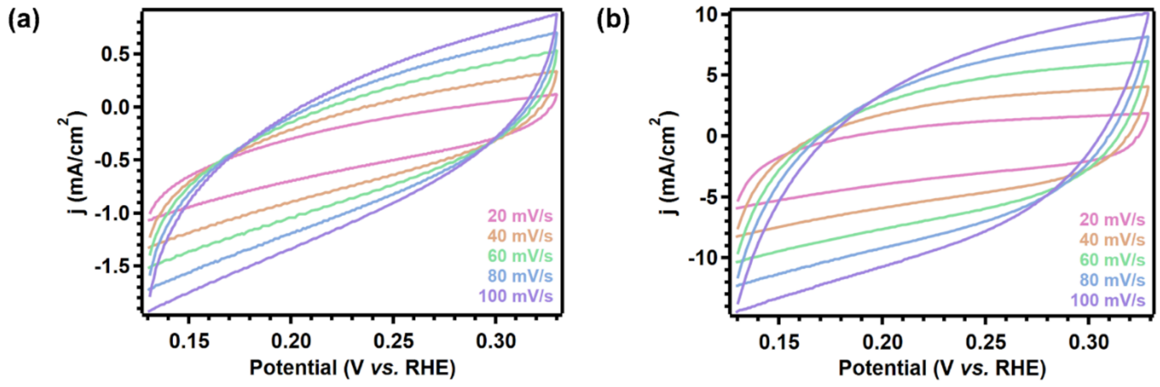

Figure S11. CV measurements were performed within a standard three-electrode configuration, where 0.5 M H_2SO_4 saturated with N_2 was used as the electrolyte. The scanning rates are 20, 40, 60, 80, and 100 mV s^{-1} respectively. **(a)** 1T-MoS_2, **(b)** Zn SAs/1T-MoS_2.

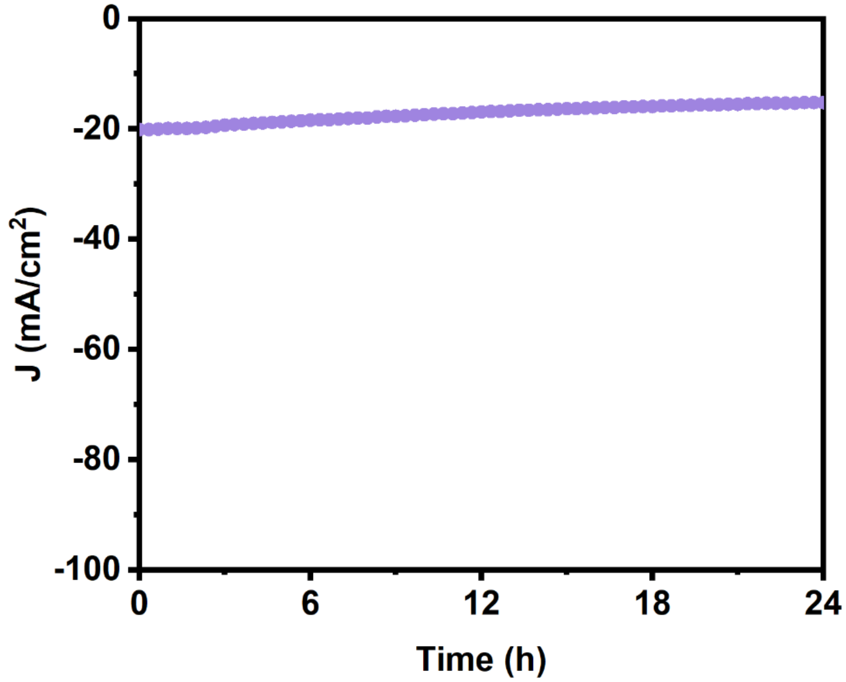

Figure S12. Recorded current generation during 24 hours of continuous electrolysis at -0.2 V *vs.* RHE with Zn SAs/1T-MoS_2 used as the working electrode.

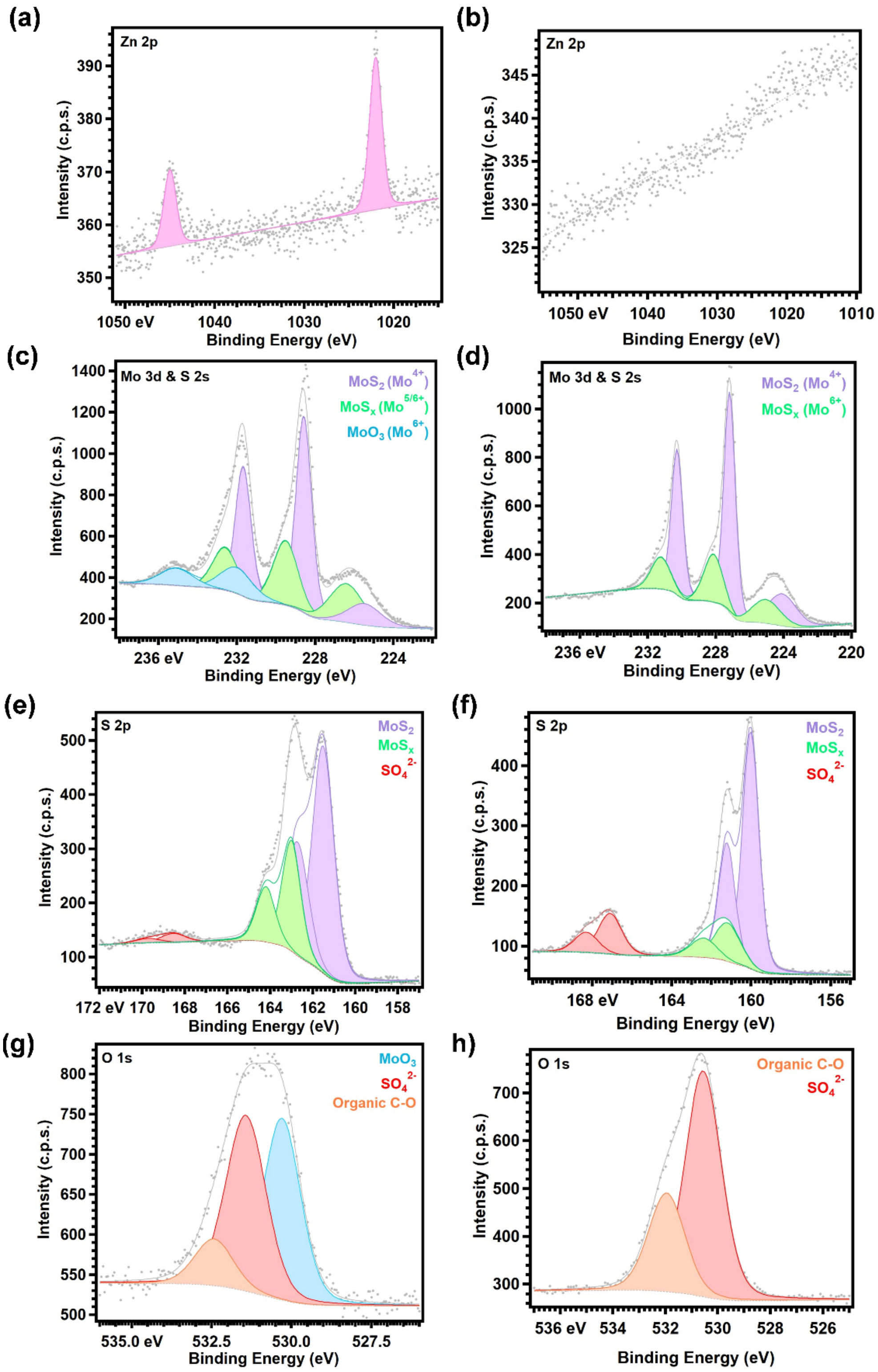

Figure S13. XPS data and peak assignments for Zn SAs/1T-MoS_2 (**a, c, e, g**) before and (**b, d, f, h**) after collecting 3,000 CV scans. (**a, b**) Zn 2p. (**c, d**) Mo 3d and S 2s. (**e, f**) S 2p. (**g, h**) O 1s.

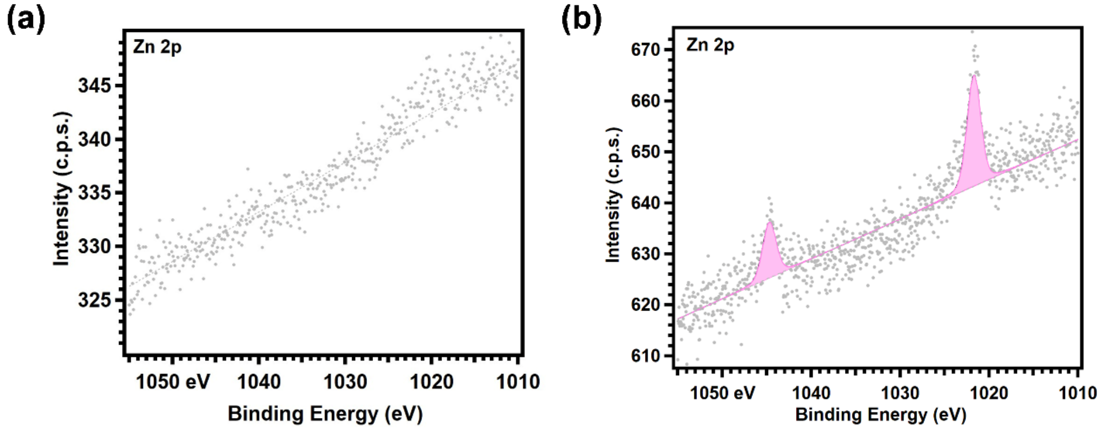

Figure S14. XPS analysis of the Zn 2p **(a)** before and **(b)** after sputtering the sample for 30 s.

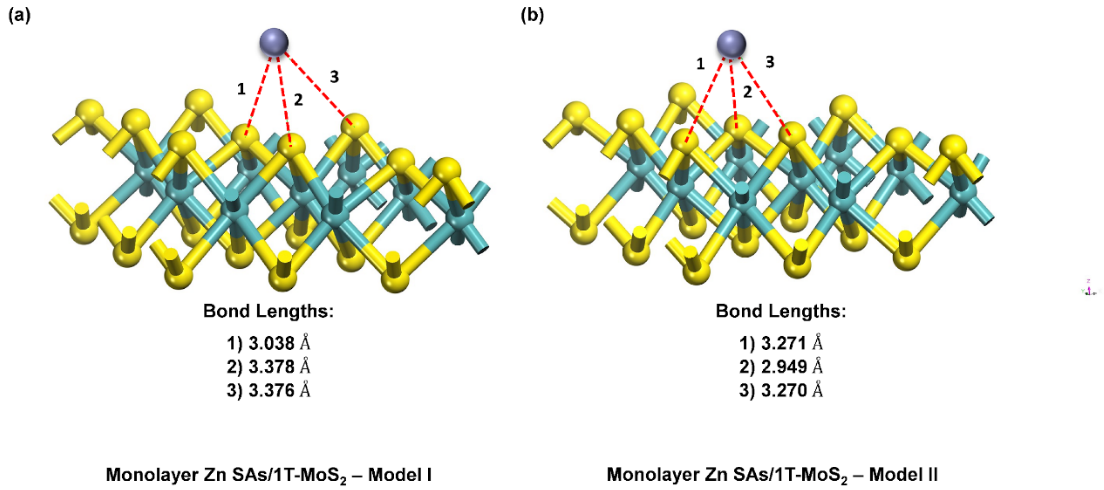

Figure S15. DFT models of the single layer Zn SAs/1T-MoS_2.

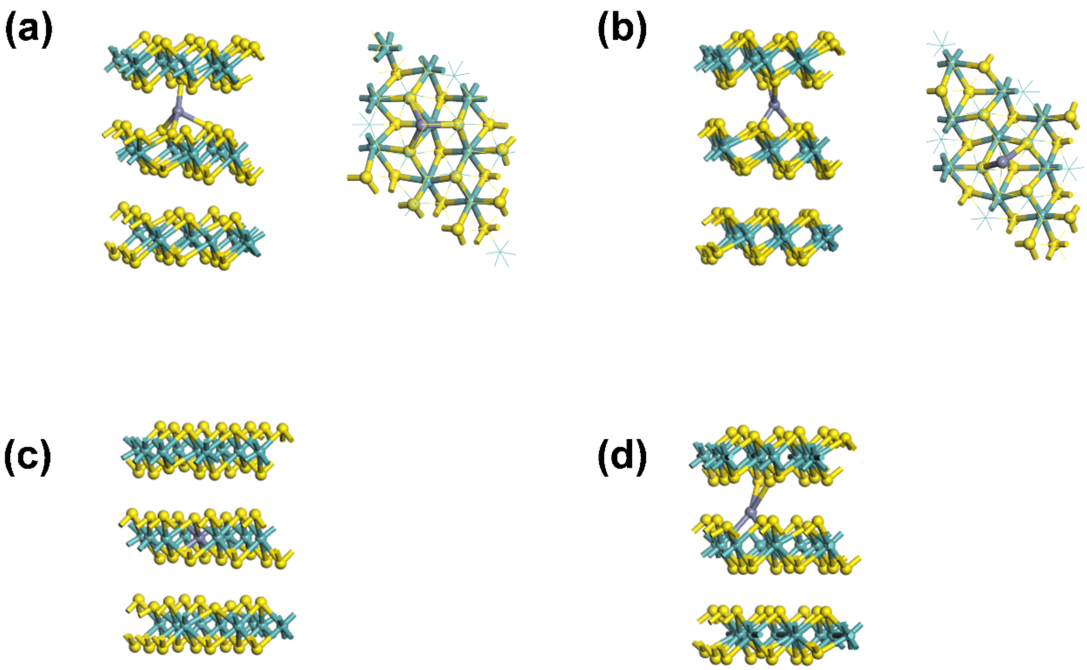

Figure S16. Possible configurations of Zn SA anchoring sites on 1T-MoS_2. **(a)** Tetrahedral coordination forming one bond with the upper basal plane and three bonds with the lower basal plane. **(b)** Tetrahedral coordination forming two bonds each with the upper and lower basal planes. **(c)** Zn substituting an Mo atom within the 1T-MoS_2 lattice. **(d)** Zn substituting an S atom in the bottom basal plane.

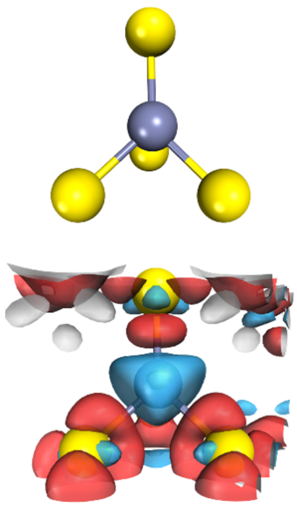

Figure S17. Mulliken charge analysis and 3D isosurface diagrams of a tetrahedrally coordinated Zn SA coordinated to S atoms along the basal plane of 1T-MoS_2. The purple and yellow atoms represent Zn and S, respectively. Charge clouds shown in blue represent energetically negative regions where electrons have been lost. Charge clouds shown in red exhibit positive charge and represent regions where electrons have been gained.

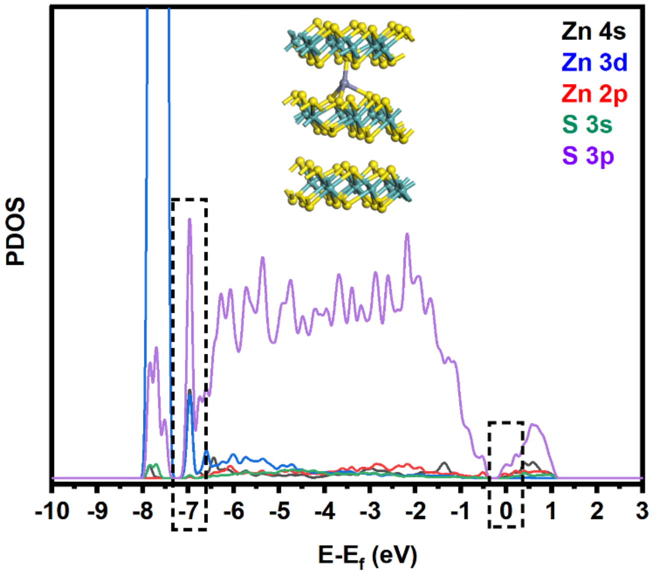

Figure S18. Partial density of states (PDOS) spectra of the Zn 4s (black), Zn 3d (blue), Zn 2p (red), S 3s (green), and S 3p (purple) orbitals.

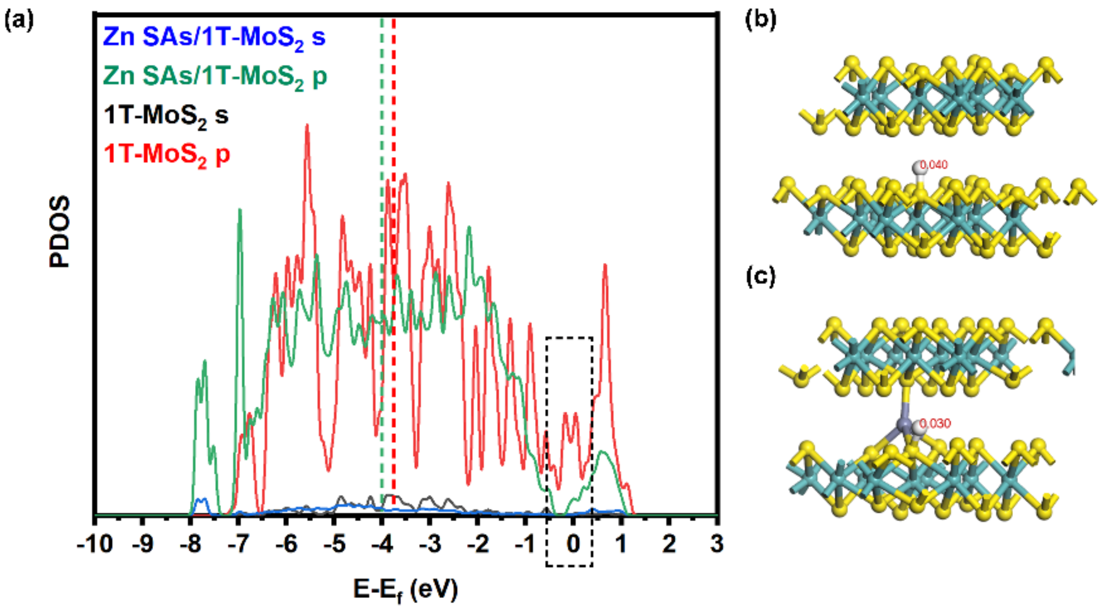

Figure S19. PDOS spectra of the (a) s and p orbitals in 1T-MoS_2 and Zn SAs/1T-MoS_2. (b) H* adsorption model on 1T-MoS_2. (c) H* adsorption model on Zn SAs/1T-MoS_2.

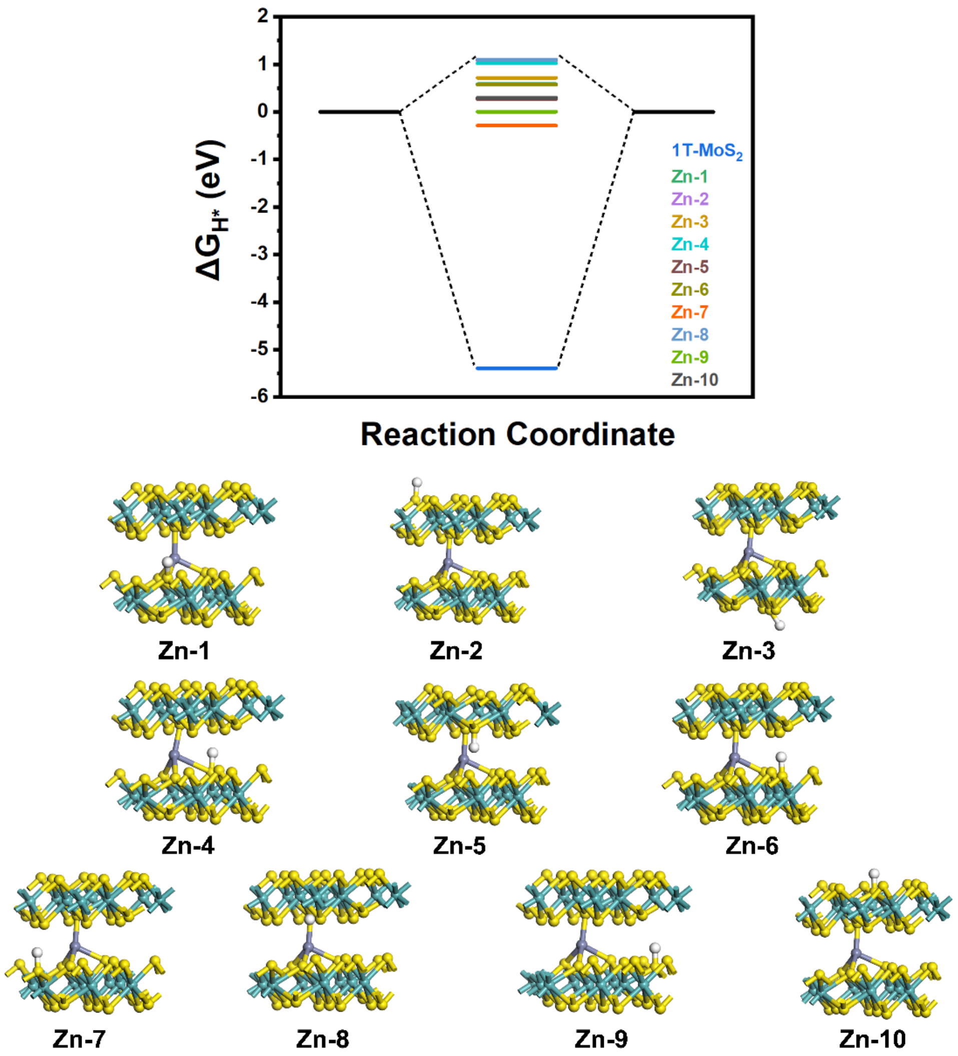

Figure S20. Calculated ΔG_{H^*} for HER at typical H* adsorption sites (at 0 V *vs*. RHE and pH = 0) with models of the H* adsorption sites Zn-1 through Zn-10.

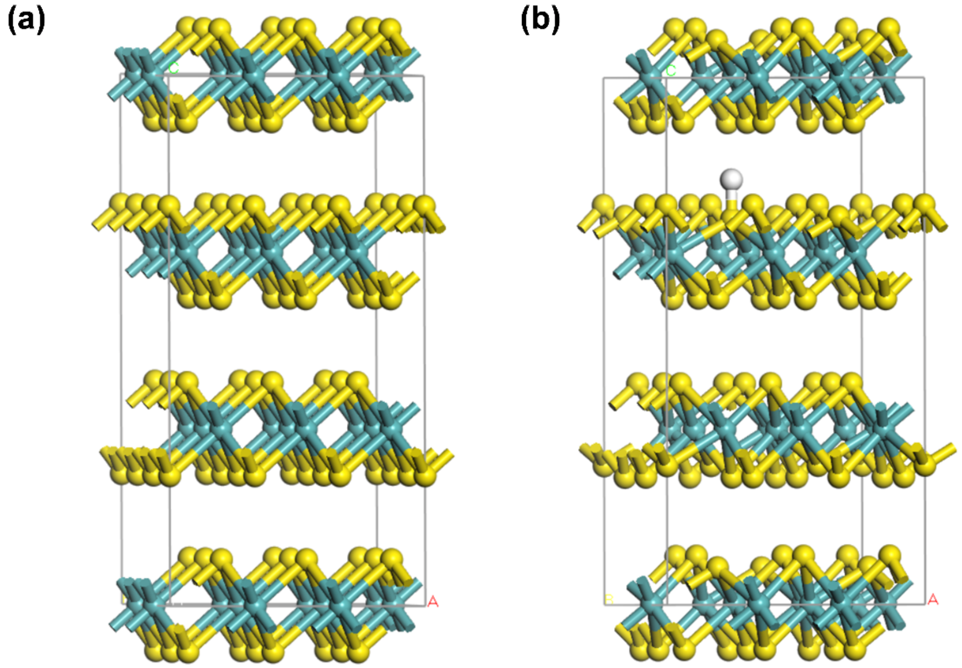

Figure S21. Supercell modeling of H* adsorption on 1T-MoS_2 with the Z axis increased by 3.4% **(a)** before and **(b)** after structural optimization.

Supplementary Table S1. XPS data and peak assignments for 1T-MoS_2 and Zn SAs/1T-MoS_2.

Peak	Binding Energy (eV)		Peak Assignment
	**1T-MoS_2**	**Zn SAs/1T-MoS_2**	
Zn $2p_{3/2}$	--	1022.80	Zn^{2+}
Zn $2p_{1/2}$	--	1045.80	Zn^{2+}
Mo $3d_{5/2}$	229.05	229.03	MoS_2, Mo^{4+}
	229.93	229.84	MoS_x, $Mo^{5/6+}$
Mo $3d_{3/2}$	232.15	232.13	MoS_2, Mo^{4+}
	233.03	232.94	MoS_x, $Mo^{5/6+}$
S $2p_{3/2}$	161.90	161.81	MoS_2, S^{2-}
	163.45	163.26	MoS_x, S^{2-}
S $2p_{1/2}$	163.10	163.01	MoS_2, S^{2-}
	164.65	164.46	MoS_x, S^{2-}
S 2s	225.95	225.93	MoS_2
	226.83	226.74	MoS_x
O 1s	531.20	531.93	Organic C=O
	532.48	533.39	Organic C-O

Supplementary Table S2. Raman peak assignments for 1T-MoS_2 and Zn SAs/1T-MoS_2.

Peak Positions (cm^{-1})		Peak Assignments
**1T-MoS_2**	**Zn SAs/1T-MoS_2**	
113	113	TA
125	124	TA
149	148	J_1
195	195	LA
213	213	LA
237	237	J_2
284	283	E_{1g}
336	334	J_3
377	376	E^1_{2g}
402	400	A_{1g}

Supplementary Table S3. Assignments of experimental g-values for 2H-MoS_2, 1T-MoS_2, and Zn SAs/1T-MoS_2.

g-values			Assignment
**2H-MoS_2**	**1T-MoS_2**	**Zn SAs/1T-MoS_2**	
	2.042	2.042	Paramagnetic S in short chains
	2.026	2.027	Electron hole center localized on S atoms
	2.019	2.017	Mo^{5+} species coordinated to S atoms
2.005	2.005	2.005	Dangling Mo-S bonds generated by S vacancies
	1.994	1.993	S-Mo^{5+} defects
	1.959	1.958	Paramagnetic Mo^{5+} coordinated to S atoms
	1.936	1.932	Mo^{5+} species coordinated to S atoms

Supplementary Table S4. Local structure parameters of Zn SAs/1T-MoS_2 fitted from the Zn K-edge EXAFS spectra.

Sample	Path	CN	ΔE (eV)	R (Å)	σ^2 (Å^2)
Zn SAs/1T-MoS_2	Zn-S	4	-9.6	2.31	0.009

Supplementary Table S5. Local structure parameters in Zn SAs/1T-MoS_2 fitted from Mo K-edge EXAFS spectra.

Sample	Path	CN	ΔE (eV)	R (Å)	σ^2 (Å^2)
1T-MoS_2 [a]	Mo-S	6	3.910	2.44	0.010
Zn SAs/1T-MoS_2	Mo-S1	3	-1.4	2.41	0.001
	Mo-S2	3	-1.4	2.62	0.0089

[a] Control sample taken from reference.[4]

Supplementary Table S6. Comparison of the overpotentials and Tafel slopes observed for each sample evaluated in this work. Overpotentials are defined as the potential (mV *vs*. RHE) measured at -10 mA/cm^2.

Sample	Overpotential (mV *vs*. RHE)	Tafel slope (mV/dec)
Carbon fiber paper (CFP)	--	--
Pt/C	37	34.0
1T-MoS_2	265	107.2
Zn SAs (2.5 mg)/1T-MoS_2	199	--
Zn SAs (8.5 mg)/1T-MoS_2	177	84.9
Zn SAs (16.5 mg)/1T-MoS_2	187	--

Supplementary Table S7. Equivalent circuit fittings for EIS data reported herein. The data was measured at overpotentials of -200 mV. R_s, R_{ct}, and CPE represent the electrolyte, charge transfer resistance, and the constant phase element, respectively.

Sample	R_s (Ω)	R_{ct} (Ω)	CPE-T	CPE-P
1T-MoS_2	2.289	127.5	0.0247	0.923
Zn SAs (2.5 mg)/1T-MoS_2	2.302	82.13	0.0573	0.828
Zn SAs (8.5 mg)/1T-MoS_2	2.191	18.41	0.00410	0.897
Zn SAs (16.5 mg)/1T-MoS_2	2.371	34.59	0.0209	0.915

Supplementary Table S8. Comparison of the HER performance of similar 1T-MoS_2 single atom catalysts. Overpotentials were measured at -10 mA/cm^2.

Catalyst	Intercalation Method	Electrolyte	Overpotential (mV *vs.* RHE)	Tafel Slope (mV/dec)	Reference
**Zn SAs/1T-MoS_2**	Adsorption	0.5 M H_2SO_4	177	84.9	This work
**Zn 2H-MoS_2**	Substitution	0.5 M H_2SO_4	300	51	*5*
**Zn 2H-MoS_2**	Substitution	0.5 M H_2SO_4	194	78	*6*
**$Ni(OH)_2$ 2H-MoS_2**	Adsorption	0.5 M H_2SO_4	139	45	*7*
**Cu_{ads} 1T-MoS_2**	Adsorption	0.5 M H_2SO_4	173	91	*8*
**Cu_{sub} 1T-MoS_2**	Substitution	0.5 M H_2SO_4	160	86	*8*
**Co 1T-MoS_2**	Adsorption	0.5 M H_2SO_4	84	47	*9*
**Mn 2H-$MoSe_2$**	Substitution	0.5 M H_2SO_4	167	60	*10*
**V 1T/2H-MoS_2**	Substitution (V^{4+}) and Adsorption (V^{2+})	0.5 M H_2SO_4	146	48	*11*

Supplementary Table S9. XPS data and peak assignments for Zn SAs/1T-MoS_2 before and after collecting 3,000 CV scans.

Peak	Zn SAs/1T-MoS_2		Peak Assignment
	Pre-CV	Post-CV	
Zn $2p_{3/2}$	1022.41	--	Zn^{2+}
Zn $2p_{1/2}$	1045.41	--	Zn^{2+}
Mo $3d_{5/2}$	229.03	228.98	MoS_2, Mo^{4+}
	229.96	229.94	MoS_x, $Mo^{5/6+}$
	232.45	--	MoO_3, Mo^{6+}
Mo $3d_{3/2}$	232.13	232.08	MoS_2, Mo^{4+}
	233.06	233.04	MoS_x, $Mo^{5/6+}$
	235.55	--	MoO_3, Mo^{6+}
S $2p_{3/2}$	161.96	161.82	MoS_2, S^{2-}
	163.45	162.99	MoS_x, S^{2-}
	168.99	168.90	SO_4^{2-}
S $2p_{1/2}$	163.16	163.02	MoS_2, S^{2-}
	164.65	164.19	MoS_x, S^{2-}
	170.19	170.10	SO_4^{2-}
S 2s	225.93	225.88	MoS_2
	226.86	226.84	MoS_x
O 1s	530.75	--	MoO_3, O^{2-}
	531.88	532.35	SO_4^{2-}
	532.88	533.75	Organic C-O

Supplementary Table S10. Inductively Coupled Plasma Optical Emission Spectroscopy (ICP-OES) analysis of 1T-MoS_2 intercalated with the various amounts of Zn SAs studied in this work.

Sample	Zn (wt%)	Mo (wt%)	Zn:Mo (mol)
Zn SAs (2.5 mg)/1T-MoS_2	0.634	29.286	0.032
Zn SAs (8.5 mg)/1T-MoS_2	1.016	27.465	0.054
Zn SAs (16.5 mg)/1T-MoS_2	1.125	24.596	0.067

REFERENCES

1. Ghatak, J.; Guan, W.; Möbus, G., In situ TEM observation of lithium nanoparticle growth and morphological cycling. *Nanoscale* **2012,** *4* (5), 1754-1759.

2. Su, C.; Tripathi, M.; Yan, Q.-B.; Wang, Z.; Zhang, Z.; Hofer, C.; Wang, H.; Basile, L.; Su, G.; Dong, M.; Meyer, J. C.; Kotakoski, J.; Kong, J.; Idrobo, J.-C.; Susi, T.; Li, J., Engineering single-atom dynamics with electron irradiation. *Sci. Adv.* **2019,** *5* (5), eaav2252.

3. Zack, L. N.; Ziurys, L. M., The pure rotational spectrum of ZnS ($X^1\Sigma^+$). *J. Mol. Spectrosc.* **2009,** *257* (2), 213-216.

4. Huang, Y.; Sun, Y.; Zheng, X.; Aoki, T.; Pattengale, B.; Huang, J.; He, X.; Bian, W.; Younan, S.; Williams, N., Atomically engineering activation sites onto metallic 1T-MoS_2 catalysts for enhanced electrochemical hydrogen evolution. *Nat. Commun.* **2019,** *10*, 982.

5. Shi, Y.; Zhou, Y.; Yang, D.-R.; Xu, W.-X.; Wang, C.; Wang, F.-B.; Xu, J.-J.; Xia, X.-H.; Chen, H.-Y., Energy level engineering of MoS_2 by transition-metal doping for accelerating hydrogen evolution reaction. *J. Am. Chem. Soc.* **2017,** *139* (43), 15479-15485.

6. Wu, W.; Niu, C.; Wei, C.; Jia, Y.; Li, C.; Xu, Q., Activation of MoS_2 Basal Planes for Hydrogen Evolution by Zinc. *Angew. Chem. Int. Ed.* **2019,** *58* (7), 2029-2033.

7. He, Z.; Liu, Q.; Zhu, Y.; Tan, T.; Cao, L.; Zhao, S.; Chen, Y., Defect-Mediated Adsorption of Metal Ions for Constructing Ni Hydroxide/MoS_2 Heterostructures as High-Performance Water-Splitting Electrocatalysts. *ACS Appl. Energy Mater.* **2020,** *3* (7), 7039-7047.

8. Li, Z.; Yan, X.; He, D.; Hu, W.; Younan, S.; Ke, Z.; Patrick, M.; Xiao, X.; Huang, J.; Wu, H.; Pan, X.; Gu, J., Manipulating Coordination Structures of Mixed-Valence Copper

Single Atoms on 1T-MoS_2 for Efficient Hydrogen Evolution. *ACS Catal.* **2022,** *12* (13), 7687-7695.

9. Qiao, W.; Xu, W.; Xu, X.; Wu, L.; Yan, S.; Wang, D., Construction of Active Orbital via Single-Atom Cobalt Anchoring on the Surface of 1T-MoS_2 Basal Plane toward Efficient Hydrogen Evolution. *ACS Appl. Energy Mater.* **2020**, *3* (3), 2315-2322.

10. Kuraganti, V.; Jain, A.; Bar-Ziv, R.; Ramasubramaniam, A.; Bar-Sadan, M., Manganese Doping of $MoSe_2$ Promotes Active Defect Sites for Hydrogen Evolution. *ACS Appl. Mater. Interfaces* **2019,** *11* (28), 25155-25162.

11. Liu, T.; Fang, C.; Yu, B.; You, Y.; Niu, H.; Zhou, R.; Zhang, J.; Xu, J., Vanadium-doping in interlayer-expanded MoS_2 nanosheets for the efficient electrocatalytic hydrogen evolution reaction. *Inorg. Chem. Front.* **2020,** *7* (13), 2497-2505.
